# Therapeutic potential of *Erxian decoction* and its special chemical markers in depression: a review of clinical and preclinical studies

**DOI:** 10.3389/fphar.2024.1377079

**Published:** 2024-06-10

**Authors:** Ning-Xi Zeng, Han Li, Meng-Yuan Su, Xin Chen, Xiao-Yan Yang, Mei Shen

**Affiliations:** ^1^ Department of Rehabilitation Medicine, People’s Hospital of Longhua, Shenzhen, China; ^2^ Institute of Biomedicine and Biotechnology, Shenzhen Institutes of Advanced Technology, Chinese Academy of Sciences, Shenzhen, China; ^3^ Department of Pharmacy, Shenzhen Bao’an Traditional Chinese Medicine Hospital, Shenzhen, China; ^4^ The First School of Clinical Medicine, Guangzhou University of Chinese Medicine, Guangzhou, China; ^5^ Department of Urology Surgery, Guangzhou Baiyun District Maternal and Child Health Hospital, Guangzhou, China

**Keywords:** Erxian decoction, depression, traditional Chinese medicine, antidepressant, review, active ingredient

## Abstract

The increasing prevalence of depression is a major societal burden. The etiology of depression involves multiple mechanisms. Thus, the outcomes of the currently used treatment for depression are suboptimal. The anti-depression effects of traditional Chinese medicine (TCM) formulations have piqued the interest of the scientific community owing to their multi-ingredient, multi-target, and multi-link characteristics. According to the TCM theory, the functioning of the kidney is intricately linked to that of the brain. Clinical observations have indicated the therapeutic potential of the kidney-tonifying formula *Erxian Decoction* (EXD) in depression. This review aimed to comprehensively search various databases to summarize the anti-depression effects of EXD, explore the underlying material basis and mechanisms, and offer new suggestions and methods for the clinical treatment of depression. The clinical and preclinical studies published before 31 August 2023, were searched in PubMed, Google Scholar, China National Knowledge Infrastructure, and Wanfang Database. This review followed the Preferred Reporting Items for Systematic Reviews and Meta-Analyses guidelines. Clinical studies have demonstrated that EXD exhibits therapeutic properties in patients with menopausal depression, *postpartum* depression, and maintenance hemodialysis-associated depression. Meanwhile, preclinical studies have reported that EXD and its special chemical markers exert anti-depression effects by modulating monoamine neurotransmitter levels, inhibiting neuroinflammation, augmenting synaptic plasticity, exerting neuroprotective effects, regulating the hypothalamic-pituitary-adrenal axis, promoting neurogenesis, and altering cerebrospinal fluid composition. Thus, the anti-depression effects of EXD are mediated through multiple ingredients, targets, and links. However, further clinical and animal studies are needed to investigate the anti-depression effects of EXD and the underlying mechanisms and offer additional evidence and recommendations for its clinical application. Moreover, strategies must be developed to improve the quality control of EXD. This review provides an overview of EXD and guidance for future research direction.

## 1 Introduction

The World Health Organization has raised concerns about the increasing incidence of depression, a psychiatric condition characterized by enduring feelings of sadness, diminished drive, despair, and the inability to experience pleasure. Depression is the third most onerous ailment worldwide and is expected to majorly contribute to the disease burden by 2030 (Malhi and Mann, 2018). The global incidence of depression was further exacerbated by the COVID-19 pandemic. Approximately 40% of Chinese adults were reported to exhibit manifestations of depressive symptoms during the COVID-19 pandemic (Qin et al., 2018; Collaborators, 2021).

The etiology of depression has not been elucidated. Early studies suggested that the downregulation of monoamine neurotransmitters contributes to the onset of depression. Thus, patients with depression are currently treated with antidepressants to augment these neurotransmitter levels (Haase and Brown, 2015). However, recent studies suggest that the etiology of depression cannot be attributed to a single mechanism. Various hypotheses have been proposed to explain the pathogenesis of depression (Blier and El Mansari, 2013; Huang et al., 2020; Tartt et al., 2022), including the neuroplasticity hypothesis, the neuroinflammation hypothesis, and the hippocampal damage hypothesis. Currently, the consensus for the treatment of depression is psychotherapy in combination with antidepressants. However, the currently used antidepressants are associated with unsatisfactory outcomes, adverse reactions, limited clinical efficacy, and poor tolerability. Hence, there is an urgent need to elucidate the pathogenesis of depression and develop efficacious antidepressant drugs.

Traditional Chinese medicine (TCM) has considerable expertise in the treatment of depression. The attributes of TCM formulations include multi-target and multi-link regulation, as well as a high safety profile (Xu et al., 2020; Zhang et al., 2022). TCM offers several advantages for the treatment of depression, such as comprehensive therapeutic approaches that address both symptomatic manifestations and underlying causes, yielding stable curative effects and preventing recurrence. According to TCM theory, the functions of the kidney and brain are correlated. Thus, the administration of kidney-tonifying formulations is beneficial for maintaining brain health, repairing brain damage, and alleviating depression.

*Erxian Decoction* (EXD), which was initially introduced by Zhang Bo-Ne in the early 1950s, is a kidney-tonifying formulation. The composition of EXD is as follows: *Epimedium brevicornu* Maxim (*Berberidaceae; Epimedii folium*; Yin Yang Huo in Chinese; 10–15 g), *Curculigo orchioides* Gaertn (*Hypoxidaceae; Curculiginis rhizoma*; Xian Mao in Chinese; 3–15 g), *Morinda officinalis* F.C.How (*Rubiaceae; Morindae officinalis radix*; Ba Ji Tian in Chinese; 10–15 g), *Angelica sinensis* (Oliv.) Diels (*Apiaceae; Angelicae sinensis radix*; Dang Gui in Chinese; 4.5–9 g), *Phellodendron chinense* C.K.Schneid. (*Rutaceae; Phellodendri chinensis cortex*; Huang Bo in Chinese; 5–15 g), and *Anemarrhena asphodeloides* Bunge (*Asparagaceae; Anemarrhenae rhizome*; Zhi Mu in Chinese; 6–15 g). EXD was developed to treat the syndromes of *kidney-yang* and *kidney-yin* deficiency, as well as to harmonize the *yin-yang* balance (Li et al., 2007; Zhang et al., 2020). *Epimedii folium* and *Curculiginis rhizoma*, which are the monarch drugs in EXD, can invigorate the *kidney-yang* and replenish the *kidney-essence* (Li et al., 2007; Wang Y. et al., 2019). *Morindae officinalis radix*, a minister drug in EXD, exerts therapeutic effects by warming and tonifying the *kidney-yang*, complementing the warming and nourishing properties of the monarch drugs (Wang Y. et al., 2019). *Anemarrhenae rhizome* and *Phellodendri chinensis cortex*, which are the assistant drugs in EXD, can nourish the *kidney-yin* and mitigate the strong and intense properties of *Curculiginis rhizoma* and *Epimedii folium* (Wang Y. et al., 2019). *Angelicae sinensis radix*, which is the envoy drug of EXD, nourishes the blood and softens the liver, facilitating blood circulation and supporting the regulatory and nourishing activities of the monarch drugs on the *Chong* and *Ren meridians* (Wang Y. et al., 2019). These six herbal medicines interact synergistically and are interconnected in their actions (Zhang et al., 2021a).

Previous phytochemical studies have demonstrated that EXD water extract contains several active ingredients, such as icariin, curculigoside, ferulic acid, berberine, timosaponin B-Ⅱ, mangiferin, quercetin, kaempferol, and luteolin (Wang N. et al., 2019; Zhang et al., 2020). According to the Chinese Pharmacopoeia (2020 edition), the threshold concentration of different bioactive components is as follows: icariin (*Epimedii folium* marker), should not be <5.0% in *Epimedii folium* decoction pieces. curculigoside (*Curculiginis rhizome* marker), should not be <0.10% in *Curculiginis rhizoma* decoction pieces; ferulic acid (*A. sinensis radix* marker), should not be <0.050% in *A. sinensis radix* decoction pieces; berberine (*Phellodendri chinensis cortex* marker), should not be <3.0% in *Phellodendri chinensis cortex* decoction pieces; timosaponin B-Ⅱ and mangiferin (*Anemarrhenae rhizome* markers), should not be <3.0% and 0.5%, respectively. Icariin, curculigoside, ferulic acid, berberine, timosaponin B-Ⅱ, and mangiferin are the special chemical markers of EXD. The concentrations of these compounds in EXD water extract serve as major parameters for assessing the quality of EXD.

The effects of EXD, which was initially developed to treat perimenopausal syndrome in women, have been examined in clinical practice and experimental studies, which have demonstrated its anti-depression potential with favorable outcomes in diverse forms of depression (Zhang et al., 2020). Moreover, preclinical studies have demonstrated that EXD exhibits distinctive advantages, such as multi-link, multi-target, and multi-pathway characteristics, as well as minimal adverse reactions. Consequently, the elucidation of the anti-depression mechanisms of EXD has clinical significance. This review provides a comprehensive summary of the clinical and preclinical studies on the anti-depression effects of EXD and its special chemical markers. Additionally, this review provides a theoretical foundation for future studies on the anti-depression effects of EXD.

## 2 Methodology

**FIGURE 1:**
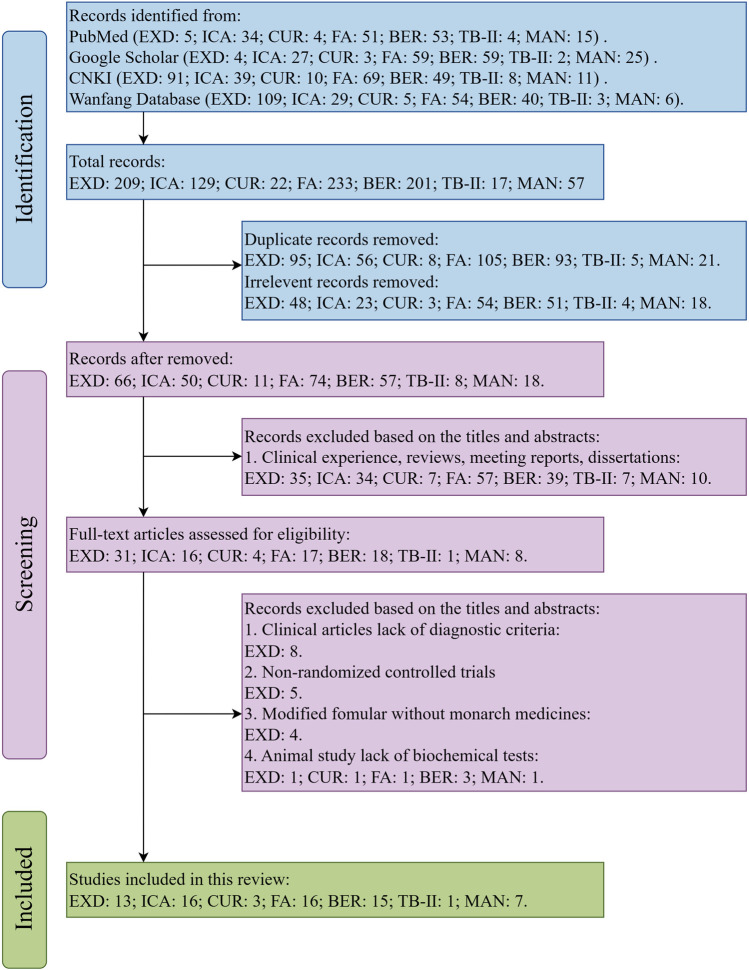
PRISMA flowchart outlining the article screening process.

This review comprehensively searched the electronic databases based on the Preferred Reporting Items for Systematic Reviews and Meta-Analyses (PRISMA) guidelines. The flow diagram of the search strategy is shown in Figure 1.

The terms “Erxian Decoction,” “Erxian Tang,” “Erxian,” “depressive disorder,” “depression,” “depressive symptom,” and “anti-depressant” and the special chemical markers of EXD were searched in the PubMed, Google Scholar, Chinese National Knowledge Infrastructure (CNKI), and Wanfang Database to retrieve studies examining the effects of EXD and its special chemical markers on depression.

The inclusion criteria employed in this study were as follows: 1) clinical and animal (*in vivo*) studies assessing the effectiveness of EXD (or its modified formula) in treating depression; 2) clinical studies that employed the Classification And Diagnostic Criteria Of Mental Disorders In China-Third-Edition (CCMD-3) or International Classification of diseases 10th Revision criteria for diagnosing patients with depression; 3) clinical studies that included a treatment group using EXD (or its modified formula) either as a standalone therapy or in conjunction with TCM formulations or antidepressants; (4) animal (*in vivo*) studies assessing the effectiveness of EXD markers in treating depression; 5) studies performed before 31 August 2023.

Meanwhile, the exclusion criteria employed in this study were as follows: 1) clinical studies that did not provide diagnostic criteria; 2) non-randomized controlled trials; 3) studies on modified formula that excluded the monarch drugs *Epimedii folium* and (or) *Curculiginis rhizoma*; 4) animal studies that lack behavioral experiments or biochemical tests; 5) studies written in languages other than Chinese and English; 6) clinical experience, reviews, meeting reports, dissertations, or meta-analysis.

## 3 Results

### 3.1 Clinical studies on the anti-depression effects of EXD

The etiology of depression has not been elucidated. Previous studies have demonstrated that the pathological process of depression is associated with various factors, including neurotransmitter dysregulation, inflammation, and neuronal impairment. EXD has been used in clinical practice for the treatment of depression in its native or modified forms in combination with other TCM formulations and conventional antidepressants. This treatment approach is effective in ameliorating depressive symptoms, yielding a favorable therapeutic effect. In this section, the studies examining the clinical anti-depression effects of EXD have been reviewed (Table 1).

**TABLE 1:** Summary of clinical studies on the anti-depression effects of EXD.

Treatment group	Control group	Treatment time	Sample size treatment/control	Diagnostic criteria	Depression type	Results	Reference
EXD (69 g, 1 dose/d) plus acupuncture (34 ± 3 years old, 3.1 ± 2.6 months of disease duration)	Maprotiline hydrochloride (75 mg/d) (35 ± 2 years old, 3.7 ± 2.3 months of disease duration)	6 weeks	37/35	CCMD-3	Postpartum depression	After treatment EPDS ↓ Serum estradiol ↑ and Treatment compared to control: treatment efficiency ↑ EPDS ↓ Serum estradiol and progesterone ↑	Li et al. (2023)
EXD plus Xiaoyaosan (114 g, 1 dose/d) (50.5 ± 10.2 years old, 31.52 ± 12.75 months of disease duration)	Paroxetine (20 mg/d) (51.3 ± 11.8 years old, 30.93 ± 11.46 months of disease duration)	8 weeks	64/63	CCMD-3	Maintenance hemodialysis depression	After treatmen HAMD ↓ (treatment and control) Serum hs-CRP ↓, TNF-α↓, and IL-6 ↓ (treatment) Treatment compared to control: treatment efficiency ↑ HAMD ↓	Huang et al. (2021)
modified EXD (122 g, 1 dose/d) plus fluoxertine (20 mg/d (48.5 ± 6.2 years old, 3.8 ± 1.2 years of disease duration)	Fluoxertine (20 mg/d) (47.8 ± 6.5 years old, 3.5 ± 1.7 years of disease duration)	8 weeks	45/45	CCMD-3	Perimenopausal depression	After treatment HAMD ↓ (treatment and control) Serum estrogen ↑ Serum LH ↓and FSH ↓ (treatment) Treatment compared to control: treatment efficiency ↑ HAMD ↓	Long (2018)
Treatment1: modified EXD (100 g, 1 dose/d) plus sertraline (50 mg/d) and psychotherapy (56.29 ± 4.61 years old, 3.22 ± 1.36 years of disease duration) Treatment2: modified EXD (100 g, 1 dose/d) plus sertraline (50 mg/d)	Sertraline (50 mg/d) (56.25 ± 11.30 years old, 3.52 ± 1.83 years of disease duration)	8 weeks	41/42/39	CCMD-3	Perimenopausal depression	After treatment SDS↓ (treatment1, 2, & control) Serum LH ↓ (treatment1), FSH ↓ (treatment1 & 2) Treatment compared to control: treatment efficiency ↑ SDS ↓	Xu et al. (2017a)
Treatment1: modified EXD (100 g, 1 dose/d) plus sertraline (50 mg/d) and psychotherapy (56.29 ± 4.61 years old, 3.22 ± 1.36 years of disease duration) Treatment2: modified EXD (100 g, 1 dose/d) plus sertraline (50 mg/d)	Sertraline (50 mg/d) (56.25 ± 11.30 years old, 3.52 ± 1.83 years of disease duration)	8weeks	41/42/39	CCMD-3	Perimenopausal depression	After treatment HAMD ↓ (treatment1, 2, & control) left occipital: 5-HT ↑ (treatment1), DA ↑(treatment1), NE (treatment2) ↑ left temporal: 5-HT ↑ (treatment1), DA ↑ (treatment1), NE ↑ (treatment1, 2, and control) Treatment compared to control: treatment efficiency ↑ (treatment1 & 2) left temporal:NE ↑ (treatment2)	Xu et al. (2017b)

**Abbreviations:** 5-HT, 5-hydroxytryptamine; CCMD-3, Classification And Diagnostic Criteria Of Mental Disorders In China-Third-Edition; DA, dopamine; EPDS, edinburgh postnatal depression scale; EXD, *erxian decoction*; FSH, follicle-stimulating hormone; HAMD, hamilton depression scale; hs-CRP, hypersensitive c-reactive protein; IL-6, interleukin-6; LH, luteinizing hormone; NE, norepinephrine; SDS, Self-rating depression scale; TNF-α, tumor necrosis factor α.

#### 3.1.1 Therapeutic effects of EXD on perimenopausal depression

Perimenopausal depression, also known as menopausal depression, is a depressive disorder that emerges during the menopausal period. In a clinical study by Xu et al. (2017b), female patients with perimenopausal depression were randomly assigned to the western medicine, integrative medicine, and integrative physical and mental treatment groups. Patients in the western medicine group were orally administered with sertraline hydrochloride (50 mg/d), while those in the integrative medicine group were orally administered with sertraline hydrochloride and modified EXD (100 g, 1 dose/d). Meanwhile, patients in the integrative physical and mental treatment group were provided psychological counseling and orally administered modified EXD and sertraline hydrochloride. After 8 weeks of treatment, the Hamilton Depression Scale (HAMD) scores in the integrative medicine group and integrative physical and mental treatment groups were lower than those in the western medicine group. Based on the HAMD score, the total effective rates were 48.89% (22/45), 78.78% (35/45), and 80.00% (36/45) in the western medicine, integrative medicine, and integrative physical and mental treatment groups, respectively. Monoamine neurotransmitter deficiency in the brain is a major etiological factor of perimenopausal depression. The administration of sertraline hydrochloride alone did not upregulate the brain levels of 5-hydroxytryptamine (5-HT), norepinephrine (NE), and dopamine (DA) in patients with perimenopausal depression. In contrast, the combination of modified EXD and sertraline hydrochloride significantly upregulated the NE levels in the left occipital region, while the combination of modified EXD, sertraline hydrochloride, and psychological counseling upregulated the levels of 5-HT and DA in both the left occipital and left anterior temporal regions. Additionally, analysis of the blood pressure, heart rate, liver and kidney functions, and electrocardiogram findings did not reveal any marked alterations (Xu et al., 2017b).

Another clinical study by Xu et al. (2017a) revealed that based on the Self-rating Depression Scale (SDS) scores, the total effective rates in the western medicine, integrative medicine, and integrative physical and mental treatment groups were 51.28%, 69.05%, and 80.49%, respectively. The ovarian function declines in perimenopausal women, leading to the dysregulation of the secretion of hormones, such as estrogen, follicle-stimulating hormone (FSH), and luteinizing hormone (LH). This interferes with the negative feedback regulation of the hypothalamic-pituitary-gonadal axis, resulting in decreased secretion of neurotransmitters by the hypothalamus. The administration of sertraline hydrochloride alone did not significantly affect the serum levels of estrogen, FSH, and LH in patients with perimenopausal depression. However, the combination of modified EXD and sertraline hydrochloride, as well as the combination of modified EXD, sertraline hydrochloride, and psychological counseling, downregulated the FSH levels. Furthermore, the combination of modified EXD, sertraline hydrochloride, and psychological counseling downregulated the LH levels.

In a study by Long, (2018), female patients with perimenopausal depression were randomly divided into the observation and control groups (45 cases/group). Patients in the control group were orally administered with fluoxetine (20 mg/d, 8 weeks), while those in the observation group were orally administered with modified EXD (122 g, 1 dose/d, 8 weeks) and fluoxetine. The HAMD scores in the observation group were lower than those in the control group. Based on the HAMD score, the total effective rates in the control and observation groups were 53.33% and 91.11%, respectively. The serum estrogen, FSH, and LH levels were not affected in the control group. In contrast, the serum levels of estrogen were upregulated and the serum levels of FSH and LH were downregulated in the observation group.

#### 3.1.2 Therapeutic effects of EXD on *postpartum* depression

*Postpartum* depression, which is characterized by the enduring presentation of depressive symptoms in women after childbirth, usually manifests within 6 weeks of childbirth. The incidence rate of *postpartum* depression is in the range of 2.1%–31.6% (Gressier et al., 2015). Furthermore, 20%–30% of patients with *postpartum* depression experience a relapse during subsequent pregnancies (Fisher et al., 2016). In a study by Li et al. (2023), 72 female patients with *postpartum* depression were randomly divided into the observation (37 cases) and western medicine groups (35 cases). Patients in the observation group were subjected to acupuncture and orally administered with EXD (69 g, 1 dose/d), while those in the western medicine group were orally administered with maprotiline hydrochloride (75 mg/d). After 6 weeks of treatment, compared with those in the western medicine group, the Edinburgh Postnatal Depression Scale scores were lower, the estrogen levels were higher, and the progesterone levels were downregulated in the observation group. The total effective rates in the observation and western medicine groups were 94.6% (35/37) and 62.9% (22/35), respectively. Furthermore, analysis of bodyweight, blood pressure, heart rate, or liver and kidney functions did not reveal marked alterations throughout the treatment period.

#### 3.1.3 Therapeutic effects of EXD on maintenance hemodialysis-associated depression

Patients undergoing maintenance hemodialysis exhibit psychological distress owing to the protracted nature of their illness, exorbitant treatment costs, and the prevalence of various severe complications. These patients are susceptible to develop negative affective states, such as anxiety and depression. In particular, depression is a prevailing mental disorder among patients undergoing maintenance hemodialysis with incidence rates in the range of 22.8%–62.0% (Griva et al., 2018; Zamanian et al., 2018). Prolonged depressive episodes adversely affect the quality of life of these patients and may increase the risk of sudden mortality. In a study by Huang et al., 2021, 130 patients with maintenance hemodialysis-associated depression were administered conventional treatment for the underlying disease and were randomly divided into control (32 male cases and 33 female cases) and observation groups (34 male cases and 31 female cases). Patients in the control group were orally administered with paroxetine (20 mg/d), while those in the observation group were orally administered with EXD and XiaoYaoSan (114 g, 1 dose/d) for 8 weeks. The total effective rates in the observation and control groups were 79.69% (51/64) and 69.84% (44/63), respectively. The HAMD scores in the observation group were lower than those in the control group. The serum levels of hypersensitive C-reactive protein (hs-CRP), interleukin (IL)-6, and tumor necrosis factor-alpha (TNF-α) were not affected in the control group but were significantly upregulated in the observation group.

### 3.2 Animal studies on the anti-depression effects of EXD

Previous studies have examined the effectiveness of EXD in ameliorating depressive symptoms and cognitive impairment in different depression models, including the maternal separation + chronic restraint stress (MS-RS)-induced, chronic unpredictable mild stress (CUMS)-induced, and reserpine-induced depression models, using a series of behavioral experiments. The findings of these experiments indicate that EXD exerts anti-depression effects by inhibiting neuroinflammation, enhancing synaptic plasticity, upregulating monoamine neurotransmitter levels, and alleviating neuronal damage. This section reviews the mechanisms through which EXD exerts anti-depression effects in the animal depression models (Table 2).

**TABLE 2:** Summary of animal studies on the anti-depression effects of EXD and the underlying molecular mechanisms.

Content (%)	Dosage	Animal	Model	Behavioral test after treatment	Molecular mechanism after treatment	Pharmacological effects	Reference
Yin Yang Huo 20 Xian Mao 20 Ba Ji Tian 20 Dang Gui 20 Huang Bo 10 Zhi Mu 10	5.85 g/kg	C57BL/6J mice	MS + RS	The central distance ↑ and central crossing ↑ in OFT	5-HT ↑, 5-HIAA ↑, DA ↑, DOPAC ↑, BDNF ↑, TrkB ↑, CREB ↑, PSD95 ↑, and SYN ↑ in hippocampus	Neurotransmitters increasement Synaptic plasticity improvement	Liang et al. (2023)
Yin Yang Huo 15 Xian Mao 10 Ba Ji Tian 16 Dang Gui 24 Huang Bo 19 Zhi Mu 16	8 g/kg	Male Wistar rats	CUMS	The sucrose preference ↑ in SPT. The distance ↑ in OFT. The immobility time ↓ in FST. The accuracy ↑ in T-maze	BrdU/DCX double-positive cells ↑ and NeuN-positive cells ↑ in hippocampus 35 differential expression proteins ↓ and 5 differential expression proteins ↑ in cerebrospinal fluid proteomics	Neuroprotection Neurogenesis promotion	Lu et al. (2022)
Yin Yang Huo 20 Xian Mao 20 Ba Ji Tian 20 Dang Gui 20 Huang Bo 10 Zhi Mu 10	1.47 g/kg	C57BL/6N mice	MS + RS	The sucrose preference ↑ in SPT. The central time ↑ and distance ↑ in OFT. The immobility time ↓ in TST. The open-arm time ↑ and crossing ↑ in EPM.	Iba-1 ↓, p-Akt1/Akt1 ↑, BDNF ↑, PSD95 ↑, and SYN ↑ in hippocampus	Anti-neuroinflammation Synaptic plasticity improvement	She et al. (2022)
Yin Yang Huo 20 Xian Mao 20 Ba Ji Tian 20 Dang Gui 20 Huang Bo 10 Zhi Mu 10	5.84 g/kg	C57BL/6J mice	MS chronic neuropathic pain	The distance ↑ and central time ↑ in OFT.	Nr3c1 ↓, GRM5 ↓, and GR ↑ in amygdala	HPA axis regulation	Zuo et al. (2022)
Yin Yang Huo 15 Xian Mao 10 Ba Ji Tian 16 Dang Gui 24 Huang Bo 19 Zhi Mu 16	8 g/kg	Male Wistar rats	Aging + CUMS	The sucrose preference ↑ in SPT. The distance ↑ and crossing ↑ in OFT. The immobility time ↓ in FST. The crossing platform times ↑, target quadrant time ↑, and distance ↑ in Morris water maze	DCX ↑, Nestin ↑, Ki-67/Nestin positive cells ↑ and BrdU/DCX positive cells ↑ in hippocampus 5 differential expression proteins ↓ and 34 differential expression proteins proteins ↑ in cerebrospinal fluid proteomics	Neuroprotection Neurogenesis promotion	Li et al. (2021)
Yin Yang Huo 20 Xian Mao 20 Ba Ji Tian 20 Dang Gui 20 Huang Bo 10 Zhi Mu 10	5.85 g/kg	C57BL/6J mice	MS + RS	The distance ↑ and central time ↑ in OFT. The open-arm time ↑ in O-maze The investigation time ↓ in SIT.	IL-6 ↓, TNF- α↓, Iba-1 ↓, and GR ↑ in hippocampus	Anti-neuroinflammation	She et al. (2021)
Yin Yang Huo 19 Xian Mao 19 Ba Ji Tian 16 Dang Gui 16 Huang Bo 15 Zhi Mu 15	0.5, 1.5, and 4.5 g/kg	Female ICR mice	OVX + CUMS	Body weight↑ The sucrose preference ↑ in SPT. The rearing ↑, grooming ↓ and defecation ↓ in OFT. The immobility time ↓ in FST and TST. The latency ↓ and target quadrant time ↑ in Morris water maze	FSH ↓, LH ↓, and IL-6 ↓ in serum BDNF ↑ and Bcl-2 ↑ in hippocampus	Neuroprotection Anti-inflammation	Zhang et al. (2021a)
Yin Yang Huo 19 Xian Mao 19 Ba Ji Tian 16 Dang Gui 16 Huang Bo 15 Zhi Mu 15	0.5, 1.5 and 4.5 g/kg	Male ICR mice	Despair model and Resepine	Despair model The immobility time ↓ in FST and TST. Reserpine The immobility time ↓ in FST and TST.	Despair model Bax ↓, Cleaved caspase-3 ↓, Caspase-8 ↓, and Bcl-2 ↑ in hippocampus Reserpine Bax ↓, Cleaved caspase-3 ↓, Caspase-8 ↓, and Bcl-2 ↑ in hippocampus 5-HT ↑, DA ↑, and NE ↑ in hypothalamic	Neuroprotection Neurotransmitters increasement	Zhang et al. (2020)

**Abbreviations:** 5-HIAA, 5-hydroxyindole acetic acid; 5-HT, 5-hydroxytryptamine; Akt, protein kinase B; Bcl-2, B-cell lymphoma-2; BDNF, brain-derived neurotrophic factor; BrdU, 5-Bromodeoxyuridinc; CREB, cAMP-response element binding protein; CSF, cerebrospinal fluid; CUMS, chronic unpredictable mild stress; DA, dopamine; DCX, doublecortin; DOPAC, dihydroxyphenylacetic acid; EPM, elevated plus maze; FSH, follicle-stimulating hormone; FST, forced swimming tests; GR, glucocorticoid receptor; GRM5, metabolic glutamate receptor 5 gene; HPA, hypothalamic-pituitary-adrenal; Iba-1, ionized calcium bindingadaptor molecule-1; IL-6, interleukin-6; LH, luteinizing hormone; MS, maternal separation; MS-RS, maternal separation combining chronic restraint stress; NeuN, neuronal nuclei; Nr3c1, nuclear receptor subfamily 3 group C member 1; OFT, open field test; PSD95, post-synaptic density protein 95; SIT, social interaction test; SPT, sucrose preference test; SYN, synaptophysin; TNF-α, tumor necrosis factor-α; TrkB, tropomyosin related kinase B; TST, tail suspension test.

#### 3.2.1 Modulation of monoamine neurotransmitter levels

The pathogenesis of depression involves multiple mechanisms, including the downregulation of monoamine neurotransmitters in the brain. NE, 5-HT, and DA are critical for the regulation of human emotions and cognitive processes. The downregulation of these neurotransmitters in the brain tissue can induce neuronal hypoactivity, leading to depression and cognitive dysfunction (Malhi and Mann, 2018). Various antidepressant medications aim to modulate the levels of these neurotransmitters. For example, selective serotonin reuptake inhibitors can augment serotonin release and concentrations in patients with depression, eliciting an antidepressant response.

Liang et al. (2023) reported that EXD upregulated the 5-HT, DA, 5-hydroxyindoleacetic acid (5-HTAA), and dihydroxyphenylacetic acid (DOPAC) levels in the hippocampus of the MS-RS-induced depression mouse model. Zhang et al. (2020) focused on the hypothalamus and established a depression mouse model by intraperitoneally injecting reserpine. The authors demonstrated that EXD upregulates the levels of 5-HT, DA, and NA in the hypothalamus of the depression mouse model. These findings suggest a correlation between the anti-depression effects of EXD and the upregulation of monoamine neurotransmitters in the brain.

#### 3.2.2 Alleviation of neuroinflammation

Recent studies have reported the upregulation of inflammatory markers in patients diagnosed with depression, suggesting a correlation between depression and neuroinflammation (Xie et al., 2023). Microglia, which are immune cells residing in the brain, maintain cerebral homeostasis and facilitate nerve restoration. Activated microglia differentiate into M1 and M2 subtypes. M1 microglia promote neuroinflammation, whereas M2 microglia exhibit anti-inflammatory properties (Wang H. et al., 2022). Ionized calcium-binding adapter molecule 1 (Iba-1) serves as a surface marker for M1 microglia. After activation, M1 microglia promote the secretion of inflammatory cytokines, including IL-1β, IL-6, and TNF-α, eliciting protective responses in the nervous system against detrimental stressors (Wang Y. L. et al., 2018). Prolonged and excessive microglial activation promotes the generation of various inflammatory mediators, resulting in neuroinflammation, exacerbation of neurotoxicity, and neuronal damage (Beurel et al., 2020; Guo et al., 2020).

She et al., 2021 demonstrated that EXD significantly decreased the mRNA and protein expression levels of Iba-1, as well as the levels of IL-6 and TNF-α in the hippocampus, in the MS-RS-induced depression mouse model. Additionally, Zhang et al. (2021b) reported that EXD downregulated the serum IL-6 contents in a perimenopausal depression mouse model.

The mitogen-activated protein kinase (MAPK) signaling pathway facilitates the proinflammatory process of microglia and is strongly correlated with impaired synaptic plasticity and neuroinflammation during the pathogenesis of depression. She et al., 2022 performed a network pharmacology analysis and reported that the MAPK signaling pathway is the principal pathway mediating the anti-depression effects of EXD. However, this finding was not experimentally verified.

#### 3.2.3 Augmentation of synaptic plasticity

Impaired synaptic plasticity has been a focus area of studies on the pathogenesis of depression. Synaptic plasticity, which encompasses alterations in the structure and function of synapses, serves as the foundation of neural plasticity. The release of mature brain-derived neurotrophic factor (BDNF) from dendrites is critical for the diverse manifestations of synaptic plasticity. BDNF binds and stimulates tyrosine receptor kinase B (TrkB) receptors, initiating the phosphatidylinositol-3-kinase (PI3K)/protein kinase B (Akt) pathway, facilitating cAMP-response element binding protein (CREB) activation, and subsequently exerting regulatory effects on synaptic plasticity (Zhang and Liao, 2020; Rana et al., 2021; Wang et al., 2021). Chronic stress is reported to downregulate the expression of BDNF and impair signal transduction. This leads to neuronal atrophy and synaptic dysfunction, diminishing stress resilience, enhancing stress susceptibility, and promoting the development of depression (Castrén and Rantamäki, 2010).

The PI3K/Akt signaling pathway enhances the viability and proliferation of neuronal cells. Additionally, the PI3K/Akt signaling pathway mediates the mechanism of action of various antidepressant medications (Zeng et al., 2022). Activated PI3K promotes the phosphorylation of Akt, which subsequently upregulates BDNF and downregulates apoptotic genes. These molecular events further contribute to the enhancement of synaptic plasticity and the maintenance of neuronal homeostasis by supporting essential processes, such as neuronutrition, neuronal survival, and the inhibition of apoptosis. Synaptophysin (SYN), a calcium-binding protein predominantly found in presynaptic terminals, serves as an indirect indicator of synaptic count, distribution, and density. Postsynaptic density protein 95 (PSD95) plays a crucial role in postsynaptic remodeling and signal transmission on the postsynaptic membrane (Li et al., 2022). Network pharmacology studies by Luo et al., 2020 and She et al., 2022 have predicted that the PI3K/Akt pathway is the major pathway mediating the anti-depression effect of EXD with Akt1 serving as the central target. In a subsequent validation experiment, She et al., 2022 demonstrated that EXD significantly upregulates the phosphorylation of Akt1 and the protein and mRNA expression levels of BDNF, PSD95, and SYN in the hippocampus of the MS-RS-induced depression mouse model.

CREB regulates various neuronal processes, including growth, development, synaptic plasticity, and the formation of long-term memory. The phosphorylation of CREB exerts beneficial effects, such as the upregulation of BDNF, the inhibition of cell apoptosis, and the facilitation of cell differentiation and repair after injury. Liang et al., 2023 demonstrated that EXD significantly enhanced the protein and mRNA levels of BDNF, TrkB, and CREB in the hippocampus of the MS-RS-induced depression mouse model. Additionally, Zhang et al. (2021a) utilized ovariectomy combined with CUMS to establish a mouse model of menopausal depression and demonstrated that EXD upregulated the BDNF levels in the hippocampus of the perimenopausal depression mouse model.

#### 3.2.4 Protection of neurons and induction of neurogenesis

Hippocampal damage is reported to be involved in the etiology of depression. Dysfunctional neurogenesis and neural loss in the hippocampus are therapeutic targets for depression (Huang et al., 2020). Cognitive impairments, including deficits in memory and learning, frequently manifest in patients with depression, indicating a strong correlation between hippocampal dysfunction and the initiation and progression of depressive symptoms. Clinical autopsies have consistently demonstrated decreased hippocampal volume along with neuronal atrophy and loss in patients diagnosed with depression. Preclinical studies have yielded empirical evidence for the reduction in the quantity and length of dendritic branches in the hippocampal neurons along with dysfunctional neurogenesis in depression animal models. Thus, the disruption of the structure and function of the hippocampus is associated with the onset and progression of depressive disorders.

Bax and B-cell lymphoma-2 (Bcl-2) proteins, which are members of the Bcl-2 family, exert pro-apoptotic and anti-apoptotic effects, respectively. Additionally, caspase-3 and caspase-8 serve as crucial initiators of apoptosis. Zhang et al., 2020 demonstrated that EXD dose-dependently upregulated Bcl-2 expression and downregulated Bax, caspase-8, and cleaved caspase-3 expression in the hippocampus of the despair mouse models and reserpine-treated mice. Additionally, another study by Zhang et al. (2021a) demonstrated that EXD dose-dependently upregulated Bcl-2 expression in the hippocampus of a menopausal depression mouse model.

Hippocampal neurogenesis encompasses the intricate mechanisms of neural stem cell proliferation and differentiation and the survival of newly formed neural cells. Previous studies have revealed a strong correlation between the dysregulation of hippocampal neurogenesis and the initiation and progression of depressive disorders. Prolonged stress impedes the proliferation of hippocampal neural stem cells, resulting in the downregulation of cell proliferation in the dentate gyrus (DG) region, which disrupts the process of neuronal differentiation and generation. Lu et al., 2022 demonstrated that EXD augmented the number of newborn precursor neurons and mature neurons in the hippocampal DG region of the CUMS-induced depression rat model. Li et al., 2021 used a combination of natural aging and CUMS to establish a rat model of late-onset depression (LOD) and demonstrated that EXD effectively increased the population of newborn neural stem cells, precursor neurons, and mature neurons in the hippocampal DG region of rats with LOD.

#### 3.2.5 Alteration of the composition of cerebrospinal fluid (CSF)

The CSF can be used for investigating central nervous system diseases as it is in direct contact with the central nervous system. Thus, CSF offers valuable insights into the physiological and pathological conditions of the brain, encompassing metabolic and biochemical reactions. The dysregulation of CSF composition promotes both physiological and pathological alterations in the brain. Positioning close to the lateral ventricle, the hippocampus is associated with the CSF. The components of the CSF can directly affect the structure and function of the hippocampus. The CSF proteome is markedly altered in patients diagnosed with depression. These differentially expressed proteins are closely linked to central nervous system damage and dysfunction in patients with depression.

Lu et al., 2022 performed proteomics analysis to identify changes in the CSF proteome of the CUMS-induced depression rat model treated with EXD. EXD mitigated the CUMS-induced dysregulation of 40 proteins in the rat CSF. These differentially expressed proteins were primarily associated with the ribosome and ubiquitin-mediated proteolysis pathway. In particular, ribosomal protein S19, ribosomal protein S12, ribosomal protein S14, vimentin, and ubiquitin-like modifier activating enzyme 1 mediated the therapeutic effects of EXD on depression and hippocampal damage. Another proteomics study by Li et al., 2021 demonstrated that EXD mitigated the CUMS-induced dysregulation of 39 proteins in the CSF of naturally aging rats. Additionally, some proteins involved in promoting neurogenesis, such as growth differentiation factor 11, neuronal cell adhesion molecule, and Ghrelin were downregulated in the CSF after CUMS modeling but were upregulated after EXD treatment.

#### 3.2.6 Regulation of the hypothalamic-pituitary-adrenal (HPA) axis

The hyperactivity of the HPA axis, a prevalent neurobiological manifestation of depression, can impair neuronal function and activity, resulting in the development of clinical symptoms of depression. Zuo et al., 2022 demonstrated that EXD upregulated glucocorticoid receptor (GR) and downregulated GR (Nr3c1) and glutamate metabotropic receptor 5 (GRM5) in the amygdala of the depression mouse model. She et al., 2021 demonstrated that EXD upregulated the expression of GR in the hippocampus of the MS-RS-induced depression mouse model.

### 3.3 Studies on the anti-depression effects of EXD special chemical markers

Icariin, curculigoside, ferulic acid, berberine, timosaponin B-Ⅱ, and mangiferin are the special chemical markers of EXD. The quantification of these components in EXD serves as a criterion for assessing EXD quality. This section primarily focuses on icariin, curculigoside, ferulic acid, berberine, timosaponin B-Ⅱ, and mangiferin as they are considered the special chemical markers that mediate the anti-depression effects of EXD (Supplementary Table S1).

#### 3.3.1 Anti-depression effects of icariin

Icariin is the most important and principal bioactive constituent in *Epimedii folium*. Previous studies have examined the effects of icariin, especially the anti-depression effects. The findings of these studies indicate that icariin is a potential candidate for the development of antidepressant medications.

Icariin is reported to exhibit anti-inflammatory properties. Previous studies have demonstrated that icariin can ameliorate depressive-like behavior in the CUMS-induced depression rat model by inhibiting the nuclear factor kappa-B (NF-κB) signaling pathway and the NOD-like receptor thermal protein domain associated protein 3 (NLRP3) inflammasome/Caspase-1/IL-1β axis, suppressing the release of TNF-α (Liu et al., 2015). Furthermore, icariin alleviates neuroinflammation in the hippocampus of mice with depression by inhibiting the high mobility group box-1 (HMGB1)/receptor for advanced glycation end-products (RAGE) signaling pathway and activating the X-box binding protein 1 spliced (XBP1s)/NF-κB signaling pathway (Liu et al., 2019). Additionally, icariin downregulates the serum levels of IL-6 and TNF-α in rats with depression (Pan et al., 2006).

The hyperactivity of the HPA axis, which is a prevalent neurobiological manifestation of depression, can impair hippocampal function and neuronal activity, resulting in the development of depression-related clinical symptoms. Icariin exerts anti-depression effects by regulating the HPA axis (Wu et al., 2011; Wei et al., 2016). Icariin can effectively downregulate the expression of corticotropin-releasing factor (CRF) in the hypothalamus and adrenocorticotropic hormone (ACTH) and corticosterone (CORT) in the pituitary gland, as well as upregulate the expression of GR in the liver, contributing to the amelioration of depressive behavior in mice (Liu et al., 2022). Furthermore, icariin downregulates the expression of GR in the hippocampus and prefrontal cortex and CRF in the serum, cortex, hippocampus, corpus striatum, and medulla oblongata in rats with depression (Pan et al., 2010; Pan et al., 2007).

Icariin exerts anti-depression effects by augmenting the monoamine neurotransmitter levels. Previous studies have demonstrated that icariin upregulates the concentrations of 5-HT, DA, and NA in the cortex, hippocampus, and striatum of the CUMS-induced depression rat model (Zhang et al., 2018). Furthermore, icariin upregulates the serum levels of 5-HT, DA, and NA in rats with perimenopausal depression (Cao et al., 2019). Additionally, the anti-depression effects of icariin include the regulation of glutamate reuptake (Zhang et al., 2017).

Icariin exerts inhibitory effects on hippocampal apoptosis in the CUMS-induced depression rat model by downregulating the expression levels of Bax, caspase-3, cleaved caspase-3, and cytochrome C and upregulating the expression of Bcl-2 (Wu et al., 2023). Moreover, icariin promotes hippocampal neurogenesis in the CUMS-induced depression rat model as evidenced by the upregulation of the number of precursor neurons and mature neurons. The icariin-mediated neurogenesis in the CUMS-induced depression rat model is closely related to alterations in CSF proteomics, especially differentially expressed proteins associated with the ribosome, PI3K/Akt, and IL-17 signaling pathways (Zeng et al., 2022). Furthermore, the icariin-mediated upregulation of BNDF and synapse activity in rats with depression involved the modulation of various proteins, including Akt, CREB, TrkB, and MAPK (Gong et al., 2016; Di et al., 2024). Additionally, the neuroprotective properties of icariin are associated with the inhibition of oxidative stress (Xue et al., 2021).

#### 3.3.2 Anti-depression effects of curculigoside

*Curculiginis rhizoma* comprises various compounds, including polysaccharides, saponins, phenols, glycosides, and terpenes. Of these, curculigoside is the sole constituent included in the Chinese Pharmacopoeia as a quality control marker for *Curculiginis rhizoma* decoction pieces.

Recent studies have provided evidence for the anti-depression properties of curculigoside. In particular, curculigoside effectively mitigates depression-like behavior in mice subjected to the learned helplessness paradigm through the upregulation of protein kinase A (PKA) pathway, the downregulation of granule cell apoptosis in the hippocampal DG region, and the inhibition of astrocyte activation (Shen et al., 2019). Alternatively, curculigoside may exert antidepressant effects by promoting the expression of hippocampal BDNF and activating the hippocampal Akt/mammalian target of rapamycin (mTOR) signaling pathway (Yang et al., 2019). Furthermore, curculigoside alleviates depression-like behaviors in the perimenopausal depression mouse model by upregulating the 5-HT and DA levels (Miao et al., 2017).

#### 3.3.3 Anti-depression effects of ferulic acid

Ferulic acid is a reliable marker for *A. sinensis radix*. Previous studies have reported that ferulic acid mitigates atypical depressive behavior in different animal models of depression, indicating that it is a potential anti-depression agent.

Studies examining the anti-depression properties of ferulic acid revealed that it exerts anti-neuroinflammatory effects (Singh et al., 2017). Ferulic acid inhibited the activation of microglia and significantly decreased the contents of IL-1β, IL-6, and TNF-α in the prefrontal cortex of the CUMS-induced depression mouse model by inhibiting the NLRP3/caspase-1/NF-κB pathway (Liu et al., 2017c). Furthermore, ferulic acid downregulates the levels of proinflammatory cytokines (such as TNF-α, IL-1β, and IL-6) and upregulates the levels of anti-inflammatory cytokines (such as IL-10) in the hippocampus of the rat depression model by inhibiting the phosphorylation of the NF-κB pathway-related proteins (Zheng et al., 2019).

Ferulic acid exerts regulatory effects on monoamine neurotransmitters. In particular, ferulic acid inhibits the reuptake of 5-HT, NE, and DA, enhancing their concentrations in different brain regions, including the hippocampus and frontal cortex (Zhang et al., 2011; Xu et al., 2013; Zhang et al., 2013; Chen et al., 2015; Sasaki et al., 2019). Ferulic acid is also reported to facilitate synaptic plasticity. Ferulic acid can upregulate the expression of BDNF and PSD95 in the prefrontal cortex and hippocampus of the depressed mouse (Yabe et al., 2010; Liu et al., 2017a), and active the CREB/BDNE/TrkB signaling pathway in the hippocampus of depressive Goto-Kakizaki rats induced by CUMS (Wang et al., 2020). Ferulic acid exerts anti-depression effects through the regulation of the HPA axis. Previous studies have reported that ferulic acid downregulates the serum ACTH and CORT levels and upregulates hippocampal GR expression in the rat depression model (Zheng et al., 2019). Additionally, the neuroprotective properties of ferulic acid are associated with the inhibition of oxidative stress, mitochondrial dysfunction, and apoptosis (Zeni et al., 2012; Lenzi et al., 2015; Zeni et al., 2017; Sasaki et al., 2019; Li et al., 2020). Furthermore, some studies have suggested a correlation between the anti-depression effect of ferulic acid and the modulation of gut microbiota (Deng et al., 2022).

#### 3.3.4 Anti-depression effects of berberine

Berberine, a prominent constituent of *Phellodendri chinensis cortex*, is a potential therapeutic for depression. Recently, berberine was reported to exert anti-depression effects in diverse animal models of depression.

Berberine effectively mitigates depression-like symptoms by exerting anti-neuroinflammation (Liu et al., 2017b and Xu et al., 2018) effects. Additionally, berberine significantly downregulated the contents of IL-1β, IL-18, pro-IL-1β, pro-IL-18, and TNF-α in the hippocampus of the CUMS-induced depression mouse model by inhibiting the activity of microglia and downregulating the expression of tripartite motif 65 (Trim65), NLRP3, caspase-1, apoptosis-associated speckle-like protein (ASC), and gasdermin D (GSDMD) (Yang et al., 2023).

Berberine significantly enhances synaptic plasticity. Previous studies have demonstrated that berberine effectively upregulates the expression of hippocampal PSD95 and SYN in mice with depression, reversing the decreased density of dendritic spines, mushroom spines, and thin spines, as well as promoting the length and depth of postsynaptic dendrites (Qin et al., 2023).

Additionally, berberine promotes the expression of monoamine neurotransmitters, upregulating the levels of 5-HT, DA, NE, and gamma-aminobutyric acid in various brain regions, including the hippocampus, cortex, striatum, and amygdala (Wang Q. et al., 2022; Ge et al., 2023).

Previous studies have reported that berberine upregulates BDNF levels, promotes neuronal survival, and stimulates neurogenesis in animals exhibiting depressive symptoms (Lee et al., 2012; Shen et al., 2016; Fan et al., 2017; Gong et al., 2019; Lu et al., 2021; Yi et al., 2021; Zhan et al., 2021; Ge et al., 2023; Qin et al., 2023). The alteration of gut microbiota has also been implicated in the anti-depression effects of berberine (Huang et al., 2023). Berberine can reverse the physical damage of gastrointestinal tract in CUMS rats (Zhu et al., 2017).

#### 3.3.5 Anti-depression effects of timosaponin B-Ⅱ and mangiferin

Saponins serve as the primary bioactive constituents in *Anemarrhenae rhizome*. Among these saponins, timosaponin B-Ⅱ constitutes 50% of the total saponin content in *Anemarrhenae rhizome*. Thus, the timosaponin B-II content is a crucial parameter for ensuring the quality control of *Anemarrhenae rhizome* decoction pieces. Timosaponin B-Ⅱ is a major bioactive compound in *Anemarrhenae rhizome*. Previous studies have demonstrated that timosaponin B-Ⅱ inhibits the reuptake of brain neurotransmitters (5-HT, NE, and DA), exerting an anti-depression effect (Lu et al., 2010).

Mangiferin can also be used to ensure the quality control of *Anemarrhenae rhizome* decoction pieces. The anti-depression properties of mangiferin are primarily attributed to its anti-inflammatory activity (Tao et al., 2023). Mangiferin treatment suppresses microglial activity and downregulates the levels of TNF-α, IL-6, and IL-1β in the hippocampus of mice with *postpartum* depression (Yan et al., 2022). Furthermore, mangiferin downregulates the contents of IL-18, IL-1β, IL-6, and TNF-α in the hippocampus of the CUMS-induced depression mouse model by inhibiting the NLRP3/ASC/caspase-1 pathway (Cao et al., 2017). Additionally, mangiferin exerts neuroprotective effects by alleviating oxidative stress levels and upregulating BDNF in the hippocampus and prefrontal cortex of mice with depression (Fu et al., 2013; Jangra et al., 2014; Luo et al., 2021).

## 4 Discussion

The prevalence of depression has recently increased. However, the currently used treatments are associated with limited efficacy and side effects and are ineffective in preventing recurrence. The pathogenesis of depression is characterized by multifactorial and intricate mechanisms. Consequently, an effective approach to treat depression should be based on multiple targets, pathways, and mechanisms. The efficacy and safety profiles of TCM formulations are higher than those of conventional prescription antidepressants owing to their multiple components and mechanisms and the ability to modulate multiple targets and pathways.

According to the basic theory of TCM, “the brain is the place where the primordial spirit resides” and “the kidney stores willpower.” “*Huangdi Neijing*,” which is the most influential classic of TCM, emphasizes that “the brain is the sea of marrow” and that “the kidney stores essence and mainly induces bones to produce marrow.” Additionally, “*Huangdi Neijing*” revealed that “if the kidney does not grow, the marrow cannot be full.” The *kidney-essence* has a critical role in maintaining diverse mental processes and emotional fluctuations. Thus, a deficiency in *kidney-essence* results in a depletion of the marrow sea, while a lack of *kidney-yang* leads to inadequate warmth nourishment and transpiration, rendering the brain susceptible to emotional and cognitive dysfunctions, including depression, anxiety, and dementia. Conversely, when the kidney is abundant in *essence* and *qi*, the marrow is adequately replenished, enhancing the resistance of the brain to illnesses. The “kidney-brain axis” theory suggests that a deficiency of kidney function plays a major role in the pathogenesis of depression and that kidney-tonifying therapy is a potential therapeutic strategy for depression.

**FIGURE 2:**
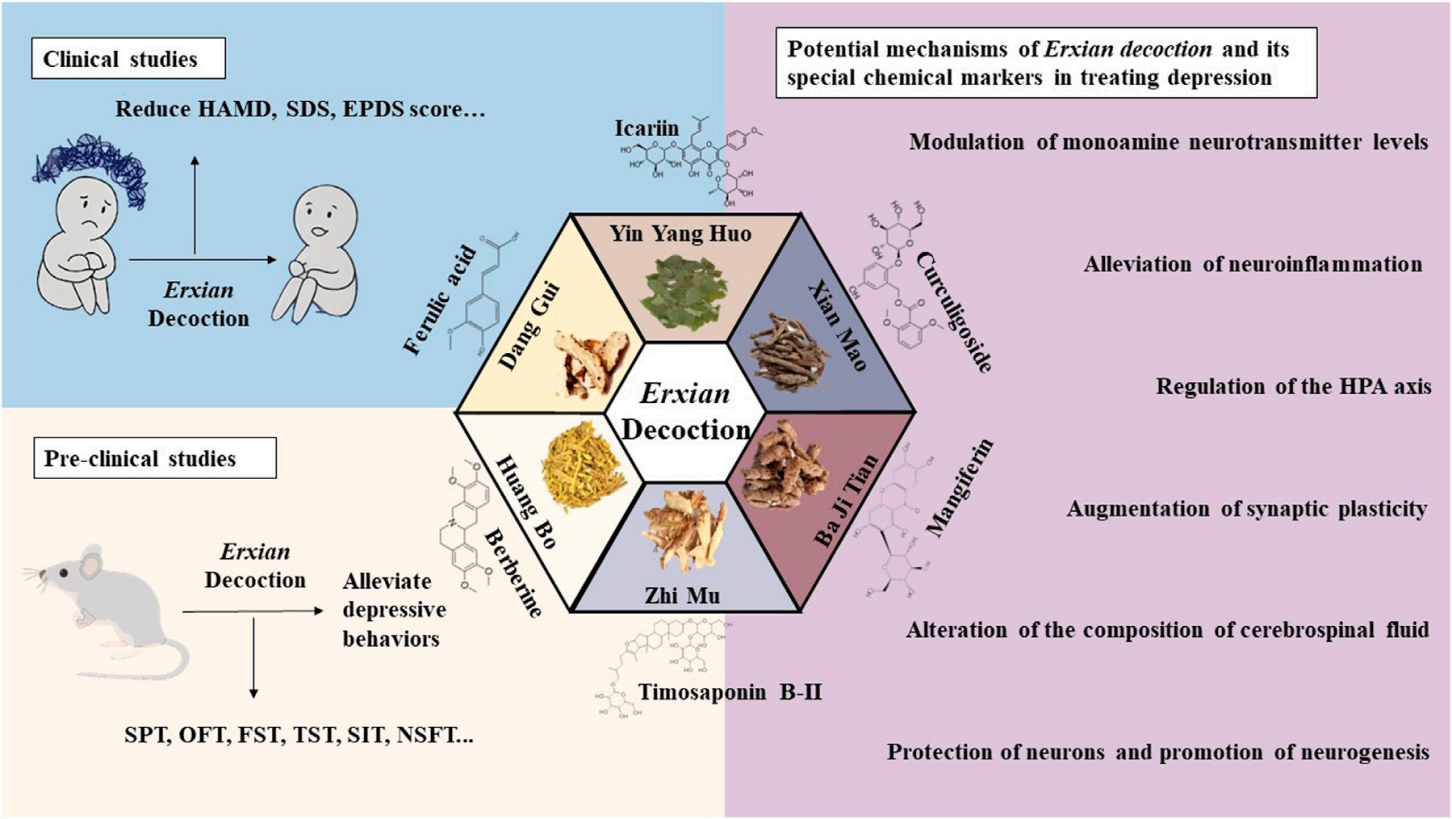
Anti-depression effects of EXD. EXD significantly decreased depression-related scores, improved the quality of life of patients, and exerted therapeutic effects on depressive disorder with limited side effects. EXD and its special chemical markers exert anti-depression effects by modulating monoamine neurotransmitter levels, inhibiting neuroinflammation, augmenting synaptic plasticity, protecting neurons, promoting neurogenesis, regulating the HPA axis, and altering the composition of CSF.

EXD, a popular kidney-toning prescription, was originally developed for the treatment of menopausal syndrome in women. Most of the previous review articles on EXD have focused on its anti-menopausal effect. This review summarized the clinical and preclinical studies on the anti-depression effect of EXD, as well as the therapeutic effects of its special chemical markers on depression, based on current research on the pathogenesis of depression (Figure 2). To the best of our knowledge, this is the first study to review the effect of EXD on depression.

Various clinical investigations have reported the efficacy of EXD in mitigating depressive symptoms among patients with depression stemming from diverse etiologies, encompassing general depression, menopausal depression, *postpartum* depression, LOD, and other depressive disorders secondary to diseases. Moreover, EXD alone or in combination with antidepressants, TCM formulations, or therapeutic modalities was efficacious in the management of depressive symptoms.

This review screened clinical studies examining the anti-depression effects of EXD. Studies lacking clear diagnostic criteria, control groups, or sufficient sample sizes were excluded from this review. These exclusion criteria were set as they indicate a lack of rigorous experimental design and limited support for the translational medicine applications of EXD to treat depression. Additionally, in clinical practice, TCM practitioners may modify the prescription based on the condition of the patient. As *Epimedii folium* and *Curculiginis rhizoma* serve as the monarch drugs in EXD, studies on modified EXD without *Epimedii folium* and *Curculiginis rhizoma* as its constituents were excluded from this review.

The results of studies that satisfied the inclusion criteria demonstrated that EXD decreases the HAMD and SDS scores, upregulates the monoamine neurotransmitter levels in the brain, and alleviates sex hormone imbalance in patients with menopausal depression. Additionally, EXD ameliorates depressive symptoms in patients with *postpartum* depression and maintenance hemodialysis-associated depression. However, these findings do not indicate that EXD is solely effective for menopausal depression, *postpartum* depression, and maintenance hemodialysis-associated depression. Several studies have reported the therapeutic effects of EXD on general depression and LOD. These studies were excluded from this review due to unclear diagnostic criteria, absence of control groups, and small sample sizes. Additionally, the optimal therapeutic regimen involving EXD must include psychotherapy. Limited numbers of clinical trials have investigated the efficacy of the combination of EXD and psychotherapy in treating depression. Thus, the existing clinical studies have provided some evidence for the therapeutic potential of EXD in depression. However, the quality of these clinical studies is not satisfactory. Hence, a large number of standardized and rigorous clinical trials must be performed to enable the application of EXD as an alternative therapy or a complementary therapy in the future.

**FIGURE 3:**
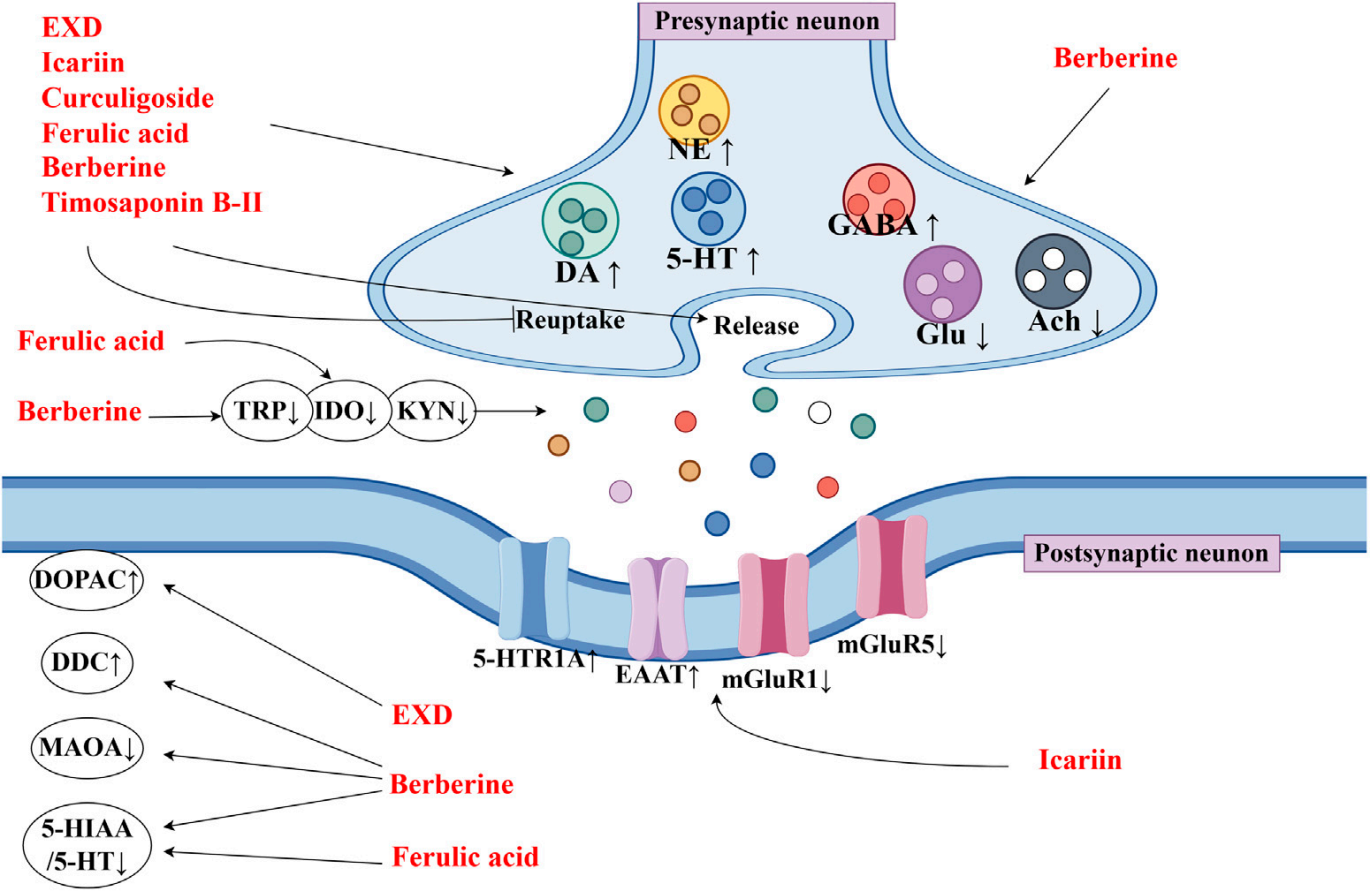
EXD modulates the monoamine neurotransmitter levels. EXD and its special chemical markers promote the synthesis of 5-HT, NE, and DA, inhibit the reuptake of 5-HT, NE, and DA, and regulate the expression of postsynaptic neurotransmitter receptors.

**FIGURE 4:**
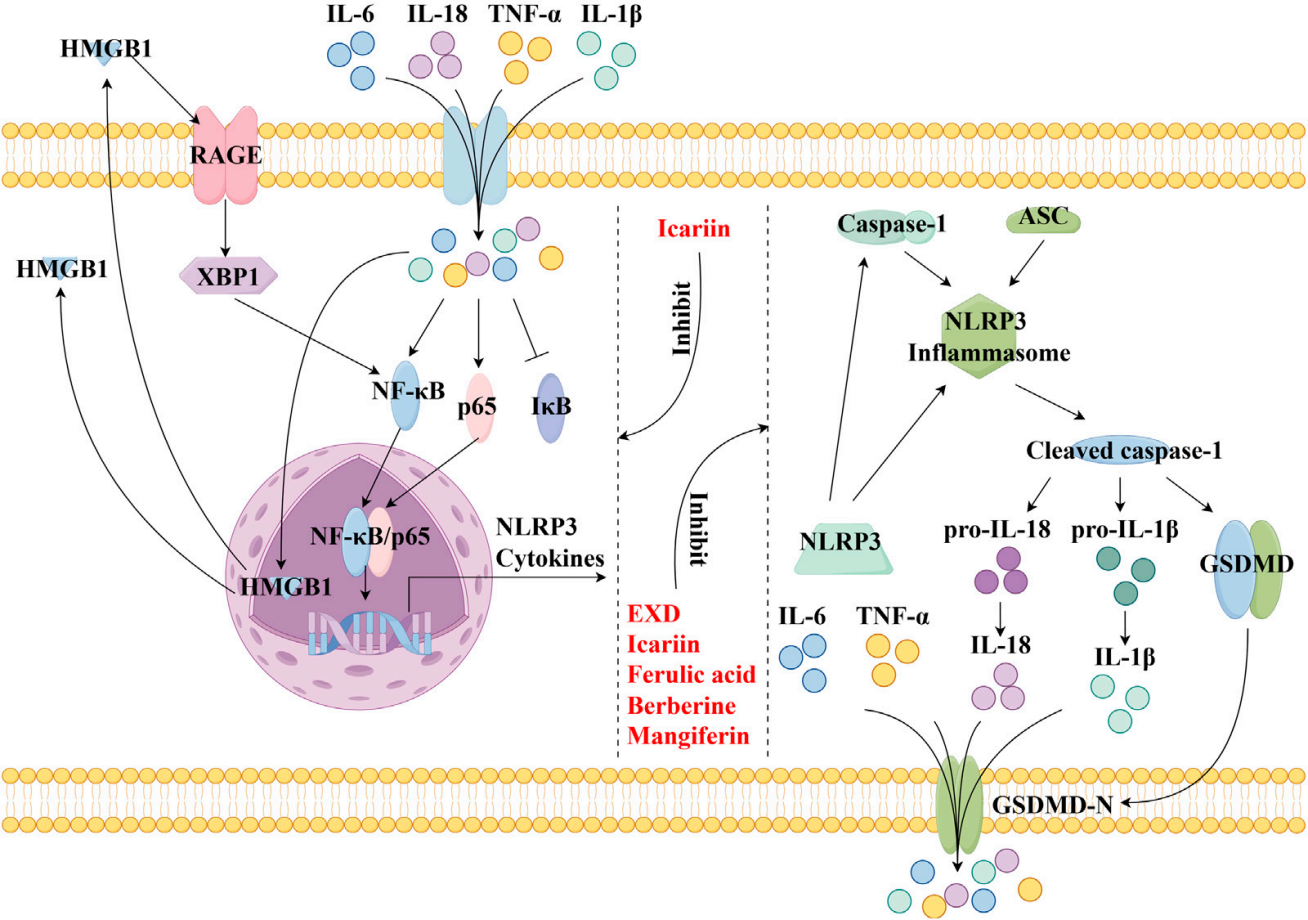
EXD inhibits neuroinflammation. EXD and its special chemical markers inhibit the NLRP3/caspase-1/IL-1β, HMGB1/RAGE, and XBP1/NF-κB pathways, suppressing the release of cytokines, such as TNF-α, IL-1β, IL-6, and IL-18.

**FIGURE 5:**
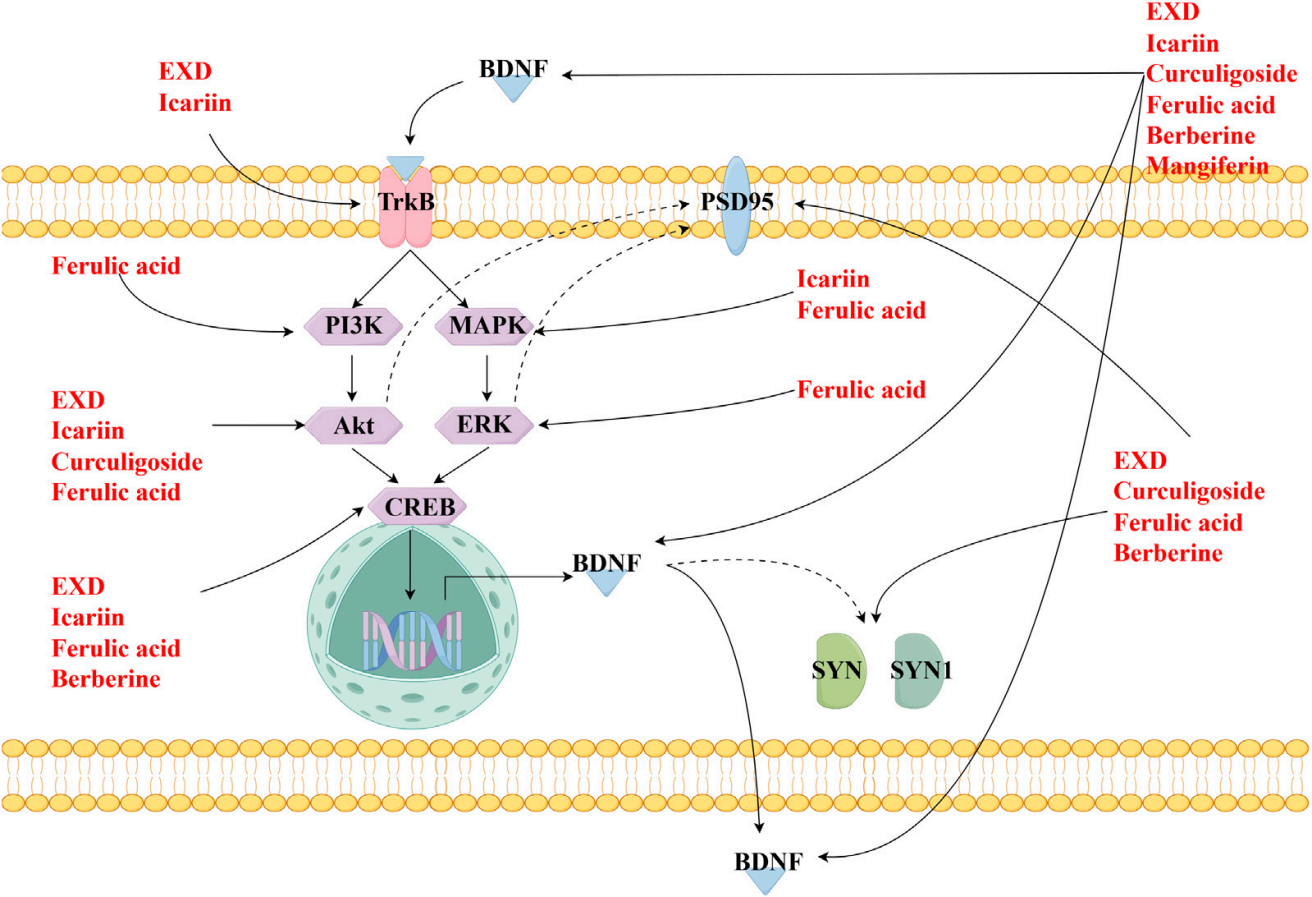
EXD augments synaptic plasticity. EXD and its special chemical markers activate the PI3K/Akt, HMGB1/RAGE, CREB/BDNF, and BDNF/TrkB pathways, promote the release of BDNF, and upregulate the expression of PSD95 and SYN.

**FIGURE 6:**
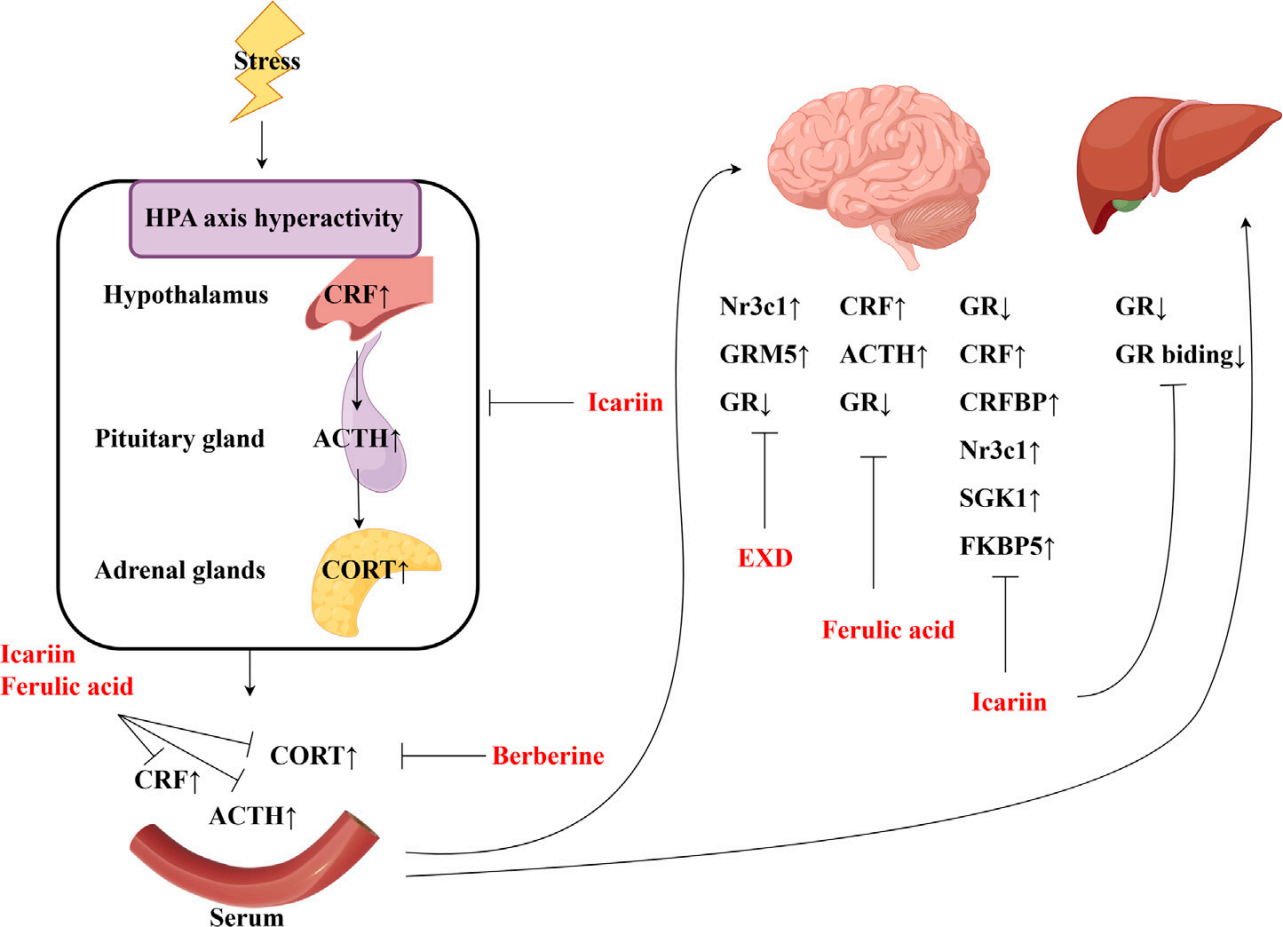
EXD regulates the HPA axis. EXD and its special chemical markers can inhibit the secretion of CRF, ACTH, and CORT, downregulate the contents of CRF, ACTH, and CORT in the blood, and regulate the expression of HPA-related hormone receptors in the brain and liver.

**FIGURE 7:**
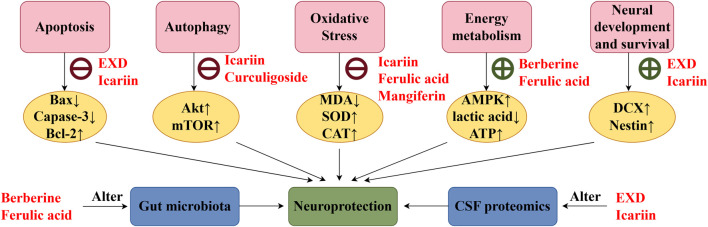
EXD exerts neuroprotective effects. EXD and its special chemical markers exert neuroprotective effects by inhibiting apoptosis, suppressing autophagy, alleviating oxidative stress, enhancing energy metabolism, promoting neurogenesis, regulating gut microbiota, and altering cerebrospinal fluid composition.

This review also screened the preclinical studies evaluating the anti-depression mechanisms of EXD. Studies lacking behavioral experiments or performing only cellular experiments were excluded as the effect of EXD on depressive symptoms cannot be determined based on these experiments. Preclinical studies have demonstrated that EXD and its special chemical markers ameliorate depressive symptoms, such as anhedonia and suppress autonomic activity and hopelessness in diverse depression models. The anti-depression effect of EXD and its special chemical markers may be attributed to the following mechanisms: 1) the modulation of monoamine neurotransmitter levels (Figure 3): EXD and its special chemical markers can promote the synthesis of 5-HT, NE, and DA, inhibit the reuptake of 5-HT, NE, and DA, and regulate the expression of postsynaptic neurotransmitter receptors; 2) the inhibition of neuroinflammation (Figure 4): EXD and its special chemical markers can inhibit the NLRP3/caspase-1/IL-1β, HMGB1/RAGE, and XBP1/NF-κB pathways to suppress the release of cytokines, such as TNF-α, IL-1β, IL-6, and IL-18; 3) the augmentation of synaptic plasticity (Figure 5): EXD and its special chemical markers can activate the PI3K/Akt, HMGB1/RAGE, CREB/BDNF, and BDNF/TrkB pathways, promote the release of BDNF, and upregulate the expression of PSD95 and SYN; 4) the regulation of the HPA axis (Figure 6): EXD and its special chemical markers can inhibit the secretion of CRF, ACTH, and CORT, downregulate the contents of CRF, ACTH, and CORT in the blood, and regulate the expression of HPA-related hormone receptors in the brain and liver; 5) exerting neuroprotective effects (Figure 7): EXD and its special chemical markers exert neuroprotective effects by inhibiting apoptosis, suppressing autophagy, alleviating oxidative stress, upregulating energy metabolism, promoting neurogenesis, regulating gut microbiota, and altering CSF composition. Several high-quality preclinical studies have revealed that EXD exerts anti-depression effects through multi-ingredient, multi-target, and multi-mechanism properties, providing strong evidence for the therapeutic application of EXD in depression.

However, further preclinical studies are needed to address some limitations. The current preclinical studies of EXD have not utilized a standardized herbal preparation, resulting in heterogeneous concentrations of active components in the water extract. The yields of icariin, curculigoside, berberine, and ferulic acid in the water extract of EXD prepared with *Epimedii folium*, *Curculiginis rhizoma*, *Morindae officinalis radix*, *A. sinensis radix*, *Phellodendri chinensis cortex*, and *Anemarrhenae rhizome* at a ratio of 9:9:9:9:6:6 were 1.605, 0.002, 1.814, and 0.007 mg/g, respectively (Wong et al., 2021). Meanwhile, the yields of icariin, curculigoside, berberine, ferulic acid, and mangiferin in the EXD water extract prepared with *Epimedii folium*, *Curculiginis rhizoma*, *Morindae officinalis radix*, *A. sinensis radix*, *Phellodendri chinensis cortex*, and *Anemarrhenae rhizome* at a ratio of 12:12:10:9:10:9 were 1.490, 0.002, 1.001, 0.1999, and 0.6591 mg/g, respectively (Cheung et al., 2017). Although all the herbs in EXD exert anti-depression effects, further studies are needed to develop a standardized herbal preparation that can enhance the effectiveness of depression treatment.

Some of the constituents of EXD are reported to exert toxic effects. The maximum oral dose of *Epimedii folium* water and alcohol extracts for mice is 80 g/kg bodyweight, which is 560 times the maximum clinical dose for humans. In long-term toxicity experiments, continuous gavage of *Epimedii folium* water and alcohol extracts at a dose of 80 g/kg bodyweight for 8 weeks significantly decreased the bodyweight of mice and dysregulated the liver and kidney function indicators in the serum. The effect of *Epimedii folium* water and alcohol extracts on the liver and kidney function indicators was mitigated at a dose of 20 g/kg bodyweight, which was 140 times the maximum dose for humans (Wang Q. et al., 2018). The maximum oral dose of *Curculiginis rhizoma* water extract for mice is 206 g/kg bodyweight, which is 1384 times the maximum clinical daily dose for humans. The half-maximal lethal dose (LD_50_) of *Curculiginis rhizoma* alcohol extract is 215.9 g/kg bodyweight, which is 1439 times the maximum clinical daily dose for humans. In acute toxicity experiments, treatment with *Curculiginis rhizoma* water extract at a dose of 206 g/kg bodyweight did not cause death in mice, while treatment with *Curculiginis rhizoma* water extract at a dose of 90 g/kg bodyweight did not exert toxic effects (Chen et al., 2021). Additionally, treatment with *Phellodendri chinensis cortex* water extract at a dose of 80 g/kg bodyweight results in acute toxicity and can cause gastrointestinal reactions (Qiu et al., 2004). The toxicity of *A. sinensis radix*, *Anemarrhenae rhizome*, and *Morindae officinalis radix* is not substantial. The conventional equivalent dose of *A. sinensis radix* is 2 g/kg bodyweight, and no acute toxic effects were observed upon treatment with *A. sinensis radix* water extract at a dose of 80 g/kg bodyweight (Min et al., 2012). The maximum oral tolerance of *Anemarrhenae rhizome* water extract for mice is 35.0 g/kg bodyweight (equivalent to 145–291 times the human dose), while that of *Anemarrhenae rhizome* alcohol extract for mice is 37.5 g/kg bodyweight (equivalent to 156–312 times the human dose) (Liu et al., 2014). Currently, the dose of EXD used in clinics is lower than that used in toxicity experiments. *Epimedii folium* can mitigate the toxicity of *Curculiginis rhizoma* (Zhu et al., 2015), while *Morindae officinalis radix* can alleviate the toxicity of *Epimedii folium* (Ling et al., 2018). Most current publications do not indicate the toxic effects of EXD. Previous studies have suggested that EXD is safe and reliable. However, long-term and high-dose oral administration of these herbal extracts may result in potential toxicity. Future studies must focus on decreasing the toxic effects of EXD. Additionally, the liver and kidney functions must be regularly monitored after the oral administration of high doses of EXD for a prolonged period.

In summary, clinical and experimental studies have reported the therapeutic potential of EXD in alleviating depression. However, several issues must be addressed. The availability of contemporary studies on the clinical utilization of EXD for depression treatment is limited with small sample sizes and diminished quality of evidence. Additionally, the optimal treatment regimen involving EXD must include psychotherapy. However, the concurrent application of EXD and psychological therapy is uncommon. Furthermore, the inadequate standardization of EXD, encompassing factors such as herbal proportion, ingredient concentration, and dose control, is a major limitation for a comprehensive analysis of the dose-response relationship. Although EXD has several targets for the treatment of depression, limited studies have identified these targets and their interconnections. All constituent herbs of EXD exhibit anti-depression properties. However, the specific component primarily mediating the anti-depression effect and the interactions among these constituents have not been elucidated. Finally, depression is a central nervous system disease. The impact of EXD on blood-brain barrier permeability has not been established.

Future studies should focus on the application of EXD, especially the combination of EXD and psychotherapy, as a therapeutic for depression by implementing multi-center, large-scale, and rigorous randomized controlled trials. Additionally, the technical barriers associated with developing standardized quality control of EXD and minimizing toxicity must be addressed to ensure the safety, efficacy, and stability of the treatment. Furthermore, contemporary methodologies, such as metabolomics, genomics, proteomics, high-performance liquid chromatography, and network pharmacology must be integrated to establish a “herbal medicine-ingredient-target-pathway” network of EXD, facilitate the elucidation of the anti-depression mechanism, and the identification of the active ingredients of EXD. These approaches will provide a theoretical foundation for the translational medicine application of EXD to treat depression.

## 5 Limitations

This study has several limitations. 1) The strict exclusion criteria of this study resulted in the non-inclusion of several studies with unsatisfactory quality. This may affect the elucidation of the therapeutic potential of EXD in depression. 2) Although nystose serves as a chemical marker of *Morindae officinalis radix*, limited studies have examined its anti-depression properties. 3) Various active ingredients of EXD, such as quercetin, luteolin, and kaempferol exhibit anti-depression properties attributed to EXD. However, these compounds are also prevalent in other herbs and are not special chemical markers of EXD. This review did not focus on these compounds.

## 6 Conclusion

Depression is a psychiatric disorder with adverse effects on the physical and mental health of patients. The etiology of depression cannot be attributed to a single mechanism. Clinical studies have demonstrated that EXD exhibits therapeutic properties in patients with menopausal depression, *postpartum* depression, and maintenance hemodialysis-associated depression, suggesting the therapeutic potential of EXD in depression. Meanwhile, experimental studies have confirmed the therapeutic effects of EXD on depression-like behavior and demonstrated its multi-ingredient, multi-target, and multi-mechanism characteristics. These studies have provided evidence for the anti-depression effects of EXD. The development of EXD as an alternative or complementary therapy for depression has a promising future. However, large-scale studies on the efficacy and side effects of EXD are lacking. Additionally, strategies to ensure the quality control of EXD are inadequate. Moreover, the anti-depression mechanisms of EXD must be further elucidated. Thus, extensive clinical and animal studies must be performed to thoroughly investigate the anti-depression effects and mechanisms, ingredients, and quality control of EXD. These studies will provide high-quality evidence and recommendations for the clinical application of EXD.

## Figures and Tables

**Table:** 

**5-HIAA**	5-hydroxyindole acetic acid
**5-HT**	5-hydroxytryptamine
**5-HTR1A**	5-hydroxytryptamine receptor 1A
**Ach**	acetylcholine
**ACTH**	adrenocorticotropic-hormone
**Akt**	protein kinase B
**AMPAR**	α-amino-3-hydroxy-5-methyl-4-isoxazolepropionic acid receptor
**ASC**	apoptosis-associated speckle-like protein
**ATP**	adenosine triphosphate
**Bcl-2**	B-cell lymphoma-2
**BDNF**	brain-derived neurotrophic factor
**BIP**	immunoglobulin heavy chain binding protein in pre-B cells
**BrdU**	5-Bromodeoxyuridinc
**CaMKII**	calcium-dependent protein kinase II
**CAT**	catalase
**CCL2**	C-C motif chemokine ligand 2
**CCMD-3**	Classification And Diagnostic Criteria Of Mental Disorders In China-Third-Edition
**CFA**	complete freund’s adjuvant
**CMS**	chronic mild stress
**CORT**	corticosterone
**CREB**	cAMP-response element binding protein
**CRF**	corticotropin releasing hormone
**CRFBP**	CRF binding protein
**CRMP2**	collapsin response mediator protein 2
**CSDS**	chronic social defeat srtress
**CSF**	cerebrospinal fluid
**CUMS**	chronic unpredictable mild stress
**DA**	dopamine
**DCX**	doublecortin
**DDC**	dopamine decarboxylase
**DG**	dentate gyrus
**DOPAC**	dihydroxyphenylacetic acid
**EAAT2**	excitatory amino acid transporter
**EGFR**	epidermal growth factor receptor
**EPDS**	Edinburgh postnatal depression scale
**EPM**	elevated plus maze
**ERK**	extracellular signal-regulated protein kinase
**EXD**	Erxian Decoction
**FKBP5**	FK506 binding protein 5
**FSH**	follicle-stimulating hormone
**FST**	forced swimming tests
**GABA**	gamma-aminobutyric acid
**GFAP**	glial fibrillary acidic protein
**Glu**	glutamate
**GR**	glucocorticoid receptor
**GRM5**	metabolic glutamate receptor 5 gene
**GSDMD-N**	gasdermin D
**GSH-Px**	glutathione peroxidase
**HAMD**	Hamilton Depression Scale
**HMGB1**	high-mobility group box 1
**HPA**	hypothalamic-pituitary-adrenal
**hs-CRP**	hypersensitive c-reactive protein
**Iba-1**	ionized calcium bindingadaptor molecule-1
**IDO**	indoleamine-2,3-dioxygenase
**IL-10**	interleukin-10
**IL-18**	interleukin-18
**IL-1β**	interleukin-1β
**IL-4**	interleukin-4
**IL-6**	interleukin-6
**iNOS**	inducible nitric oxide synthase
**LPS**	lipopolysaccharides
**IκB**	inhibitor of NF-κB
**KYN**	kynurenine
**LH**	luteinizing hormone
**LHS**	learned helplessness stress
**LOD**	late-onset depression
**MAOA**	monoamine oxidase A
**MAP-2**	microtubule associated protein-2
**MAPK**	mitogen-activated protein kinase
**MDA**	malondialdehyde
**mGluR1**	metabotropic glutamate receptor 1
**mGluR5**	metabotropic glutamate receptor 1
**MS-RS**	maternal separation combining chronic restraint stress
**mTOR**	mechanistic target of rapamycin
**NE**	norepinephrine
**NeuN**	neuronal nuclei
**NF-κB**	nuclear factor kappa-B
**NLRP3**	NOD-like receptor thermal protein domain associated protein 3
**NMDAR**	N-methyl-D-aspartate receptor
**nNOS**	neuronal nitric oxide synthase
**NO**	nitric oxide
**Nr3c1**	nuclear receptor subfamily 3 group C member 1
**NSFT**	novelty suppressed feeding test
**OFT**	open field test
**OVX**	ovariectomized
**PI3K**	phosphatidylinositide 3-kinases
**PKA**	protein kinase A
**PKC**	protein kinase C
**PPD**	perimenopausal depression
**PRS**	prenatal restraint stress
**PSD95**	post-synaptic density protein 95
**RAGE**	receptor for advanced glycation end-products
**SDS**	Self-rating depression scale
**SGK**	glucocorticoid-inducible kinase
**SIRT6**	sirtuin 6
**SOD**	superoxide dismutase
**SIT**	social interaction test
**SPT**	sucrose preference test
**SYN**	synaptophysin
**TCM**	Traditional Chinese Medicine
**TLR4**	toll-like receptor 4
**TNF-α**	tumor necrosis factor-α
**TPH1**	tryptophan 5-hydroxylase 1
**Trim65**	tripartite motif-containing protein 65
**TrkB**	tropomyosin related kinase B
**TRP**	tryptophan
**TST**	tail suspension test
**XBP1**	X-box binding protein 1
