# Supplementary material for: Therapeutic potential of Erxian decoction and its special chemical markers in depression: a review of clinical and preclinical studies
Source: Front Pharmacol. 2024 Jun 10;15:1377079. doi: 10.3389/fphar.2024.1377079 (PMC11194323; doi:10.3389/fphar.2024.1377079)
Supplement: Supplementary file 1 [file Table1.pdf]

Table S1. Summary of the antidepressant-like effects and its mechanisms of the core active ingredients of EXD

Drug	Dosage	Inducement	Animal	Behavioral test after treatment	Molecular mechanism after treatment	Pharmacological effects	Reference
Icariin	20 mg/kg	CUMS	Male SD rats	The sucrose preference in SPT ↑.<br>The center time and frequency in OFT ↑.<br>The open arms time and frequency in EPM ↑.<br>The immobility time in FST ↓.	Apoptosis cells ↓, Bax ↓, Cleaved caspase-3 ↓, Caspase-3 ↓, Cytochrome C ↓, Bcl-2 ↑, and GR ↓ in hippocampus.	Neuroprotection	(Wu et al., 2023)
Icariin	10, 20 and 40 mg/kg	CUMS	Male ICR mice	The sucrose preference in SPT ↑.<br>The uprights and crossings in OFT ↑.<br>The immobility time in FST and TST ↓.	5-HT ↑, DA ↑, NE ↑, TNF-α ↓, IL-6 ↓, IL-1β ↓, BDNF ↑, p-Akt/Akt ↑, p-CREB/TrkB ↑, MAPK3 ↑, MAPK1 ↑, Bcl-2 ↑, EGFR ↑, and mTOR ↑ in brain.	Neurotransmitters modulation<br>Anti-neuroinflammation<br>Synaptic plasticity improvement<br>Neuroprotection	(Di et al., 2023)
Icariin	120 mg/kg	CUMS	Male Wistar rats	The sucrose preference in SPT ↑.<br>The distance and crossings in OFT ↑.<br>The immobility time in FST ↓.<br>The accuracy in T-maze ↑.	BrdU/DCX double-positive cells ↑, and NeuN-positive cells ↑ in hippocampus.<br>44 differential expression proteins ↓ and 8 differential expression proteins ↑ in cerebrospinal fluid proteomics.	Neurogenesis promotion<br>Neuroprotection	(Zeng et al., 2022)
Icariin	30, 60 mg/kg	CSDS	Male C57 mice	The crossings in OFT ↑.<br>The social time in SIT ↑.	CRF ↓ in hypothalamus.<br>ACTH ↓ in pituitary gland.<br>CORT ↓ in adrenal gland.<br>GR ↑ and GR binding assay ↑ in liver.<br>IL-6 ↓ and TNF-α ↓ in serum.	HPA axis regulation<br>Anti-inflammation	(Liu et al., 2022)
Icariin	20 mg/kg	CUMS	Male SD rats	The distance and stand-up in OFT ↑.<br>The immobility time in FST ↓.<br>The latency ↑ in Morris water maze.	MDA ↓, NO ↓, SOD ↑, and GSH-pX ↑ in serum.<br>TNF-α ↓, IL-1β ↓, IL-6 ↓, IL-10 ↑, Bax ↓, Caspase-9 ↓, Caspase-3 ↓, and Bcl-2 ↑ in hippocampus	Neuroprotection<br>Anti-neuroinflammation	(Xue et al., 2021)

Drug	Dosage	Inducement	Animal	Behavioral test after treatment	Molecular mechanism after treatment	Pharmacological effects	Reference
Icariin	12.5, 25 and 50 mg/kg	PPD	Female SD rats	The distance and stand-up in OFT ↑.<br>The immobility time in FST ↓.	5-HT ↑, DA ↑, and NE ↑ in serum.	Neurotransmitters modulation	(Cao et al., 2019)
Icariin	20 mg/kg	CSDS	Male C57BL/6 J mice	The social avoidance in SIT ↓.<br>The center time in OFT ↑.<br>The open arms time in EPM ↑.	TNF-α in serum ↓.<br>Total HMGB1 ↑, total p65 ↑, cytoplasmic HMGB1 ↓, nuclear HMGB1 ↑, nuclear p65 ↑, BIP ↓, XBP1s ↓, pIκB ↑, IκB ↑, RAGE ↓, and MAP-2 ↑in hippocampus.	Anti-neuroinflammation	(Liu et al., 2019)
Icariin	10, 20 and 40 mg/kg	CUMS	Male SD rats	The sucrose preference in SPT ↑.<br>The crossings and rearings in OFT ↑.<br>The immobility time in FST ↓.	5-HT ↑, DA ↑, and NE ↑ in prefrontal cortex, corpus striatum and hippocampus.<br>5-HT ↑ in corpus striatum and hippocampus.	Neurotransmitters modulation	(Zhang et al., 2018)
Icariin	40 and 80 mg/kg	PRS	Male SD rats	The sucrose preference in SPT ↑.<br>The immobility time in FST ↓.	mGluR1 ↓, mGluR5 ↓, and EAAT2 ↑ in hippocampus.	Neuroprotection	(Zhang et al., 2017)
Icariin	20 and 40 mg/kg	CUMS	Male SD rats	The sucrose preference in SPT ↑.	Cytosolic GR ↓, cytosolic SGK1 ↓, and FKBP5 ↓ in hippocampus.<br>Cytosolic GR ↓, cytosolic SGK1 ↓, nuclear GR ↓, FKBP5 ↓, and Nr3c1 ↓ in prefrontal cortex.	HPA axis regulation	(Wei et al., 2016)
Icariin	60 mg/kg	CORT	Male SD rats	The sucrose preference in SPT ↑.<br>The immobility time in FST ↓.	BDNF ↑ in hippocampus.<br>VLDL/LDL ↓, Unsaturated lipids ↓, pyruvate ↑, N-Acetyl glycoproteins ↓, Creatine/Phosphocreatine ↑, Glucose ↑, and Phenylalanine ↑ in serum metabonomic.<br>Succinate ↓, 2-Oxoglutarate ↓, Taurine ↑, and Formate ↓ in urine metabonomic.<br>N-Acetylaspartate ↑, Aspartate ↓, and Taurine ↑ in brain metabonomic.	BDNF increasement<br>Serum metabonomic alternation<br>Brain metabonomic alternation	(Gong et al., 2016)

Drug	Dosage	Inducement	Animal	Behavioral test after treatment	Molecular mechanism after treatment	Pharmacological effects	Reference
Icariin	20 and 40 mg/kg	CUMS	Male SD rats	The sucrose preference in SPT ↑.<br>The immobility time in FST ↓.	iNOS ↓, MDA ↓, NO ↓, SOD ↑, CAT ↑, NLRP3 ↓, NF-κB ↓, Caspase-1 ↓, IL-1β ↓, and TNF-α ↓ in hippocampus.	Anti-neuroinflammation<br>Neuroprotection	(Liu et al., 2015)
Icariin	25 and 50 mg/kg	CSDS	Male C57BL/6 J mice	The social interaction ↑ in SIT.	IL-6 ↓ and CORT ↓ in serum.<br>GR ↑, GR binding assay ↑ in liver.	HPA axis regulation	(Wu et al., 2011)
Icariin	30 and 60 mg/kg	CMS	Male Wistar rats	The sucrose intake ↑ in SPT.	CRF ↓ and CORT ↓ in serum.<br>CRF ↓, pCREB/CREB ↑, CREB ↑, 5-HTR1A ↑, and GR ↑ in hippocampus.<br>CRF ↓ and CRFBP ↓ in hypothalamus.<br>CRF ↓, CRFBP ↓, pCREB/CREB ↑, and 5-HTR1A ↑ in frontal cortex.	HPA axis regulation<br>Neuroprotection	(Pan et al., 2010)
Icariin	15, 30 and 60 mg/kg	CMS	Male Wistar rats	The sucrose intake ↑ in SPT.	CRF ↓ in serum, cortex, hippocampus, corpus striatum, medulla oblongata, and hypothalamus.	HPA axis regulation	(Pan et al., 2007)
Icariin	35 and 70 mg/kg	CMS	Male SD rats	The sucrose intake ↑ in SPT.	CRF ↓, CORT ↓, TNF-α ↓, and IL-6 ↓ in serum.	HPA axis regulation<br>Anti-inflammation	(Pan et al., 2006)
Curculigoside	1.6, 8 and 40 mg/kg	LHS	Male C57BL/6 mice	The immobility time ↓ in FST and TST.	Apoptosis cells ↓, GFAP ↑, pPKA↑, and PSD95 ↑ in hippocampus.	Synaptic plasticity improvement<br>Neuroprotection	(Shen et al., 2019)
Curculigoside	1.6, 8 and 40 mg/kg	LHS	Male C57BL/6 mice	The immobility time ↓ in FST and TST.<br>The target quadrant time ↑ in Morris water maze.	BDNF ↑, p-Akt/Akt ↑, and p-mTOR/mTOR ↑ in hippocampus	Neuroprotection	(Yang et al., 2019)
Curculigoside	100, 200 and 400	PPD	Female Kunming	The immobility time ↓ in FST and TST.	5-HT ↑ and DA ↑ in brain.	Neurotransmitters modulation	(Miao et al., 2017)

Drug	Dosage	Inducement	Animal	Behavioral test after treatment	Molecular mechanism after treatment	Pharmacological effects	Reference
	mg/kg		mice				
Ferulic acid	20 mg/kg	LPS	Male C57BL/6J mice	The sucrose preference ↑ in SPT.<br>The distance ↑ and center time ↑ in OFT.<br>The open arms time ↑ and open arms frequency ↑ in EPM.<br>The immobility time ↓ in FST.	Firmicum/Bacteroidetes ↑, Firmicutes ↑, Verrucomicrobia ↓, Adlercreutzia ↑, Parabacteroides ↓, Solibacillus ↑, Acinetobacter ↑, Arthrobacter ↑ in gut microbiota essay.<br>5-hydroxytryptophan ↑, 20-carboxy-leukotriene B4 ↑, N-methylhistamine ↓, D-sphingosine ↓, and Phosphatidylcholine ↓ in microbial metabolomic.	Gut microbiota alternation<br>Microbial metabolomic alternation	(Deng et al., 2022)
Ferulic acid	40 and 80 mg/kg	CUMS	Male C57BL/6J mice	The sucrose preference ↑ in SPT.<br>The immobility time ↓ in FST and TST.	SIRT6 ↓, pAKT ↑, and pCRMP2 ↓ in hippocampus.	Neuroprotection	(Li et al., 2020)
Ferulic acid	1.36 mg/kg	CUMS	Male Goto-Kakizaki rats	The immobility time ↓ in FST and TST.<br>The distance ↑ in OFT.	BDNF ↑ in serum.<br>CREB ↑, BDNF ↑, and TrkB ↑ in hippocampus.	Neuroprotection	(Wang et al., 2020)
Ferulic acid	12.5, 25, and 50 mg/kg	Prenatal stress	Male offspring SD rats	The sucrose preference ↑ in SPT.<br>The immobility time ↓ in FST.<br>The center crossings ↑, total crossings ↑, rearing ↑, and grooming ↑ in OFT.	ACTH ↓ and CORT ↓ in serum.<br>Nissl-positive cells ↑, nNOS ↓, TNF-α ↓, IL-1β ↓, IL-6 ↓, IL-10 ↑, p-NF-κB/NF-κB ↓, and GR ↑ in hippocampus.	HPA axis regulation<br>Anti-neuroinflammation<br>Neuroprotection	(Zheng et al., 2019)
Ferulic acid	5 mg/kg	TST	Male ICR mice	The immobility time ↓ in TST.	Energy metabolism ↑, cell proliferation ↑, and dopaminergic synthesis related signaling pathway genes ↑ in brain microarray analysis.<br>DA ↑, NE ↑, BDNF ↑, ATP ↑, and glycogen ↓ in brain.<br>CORT ↓ in serum.	Neurotransmitters modulation<br>Neuroprotection	(Sasaki et al., 2019)

Drug	Dosage	Inducement	Animal	Behavioral test after treatment	Molecular mechanism after treatment	Pharmacological effects	Reference
Ferulic acid	1 mg/kg	CORT	Male Swiss mice	The immobility time ↓ in FST.	Brain:<br>MDA ↓, protein carbonyl ↓nitrite ↓, and non-protein thiol groups↑<br>MDA ↓ and nitrite ↓ in serum.	Neuroprotection	(Zeni et al., 2017)
Ferulic acid	20, 40 and 80 mg/kg	CUMS	Male ICR mice	The sucrose preference ↑ in SPT.<br>The immobility time ↓ in TST.	IL-1β↓, IL-6 ↓, TNF-α ↓, CD11b ↓, pNF-κB ↓, pNF-κB/NF-κB ↓, NLRP3 ↓, and Caspase-1 ↓ in prefrontal cortex.<br>IL-1β ↓ in serum.	Anti-neuroinflammation	(Liu et al., 2017c)
Ferulic acid	20 and 40 mg/kg	CUMS	Male ICR mice	The sucrose preference ↑ in SPT.<br>The immobility time ↓ in FST.	BDNF ↑ and PSD95 ↑ in both hippocampus and prefrontal cortex.	Synaptic plasticity improvement	(Liu et al., 2017a)
Ferulic acid	40 and 80 mg/kg	TST	Male Swiss mice	The immobility time ↓ in TST.	IL-1β ↓, TNF-α ↓, and IDO ↓ in brain.<br>CORT ↓ in serum.	Anti-neuroinflammation<br>HPA axis regulation	(Singh et al., 2017)
Ferulic acid	0.01, 0.1, 1 and 10 mg/kg	TST and FST	Male Swiss mice	The immobility time ↓ in TST and FST.	SOD ↑, CAT ↑, GSH-Px↑, and MDA ↓ in both blood, hippocampus, and cerebral cortex.	Neuroprotection	(Lenzi et al., 2015)
Ferulic acid	10, 20, 40 and 80 mg/kg	TST and FST	Male ICR mice	The immobility time ↓ in TST and FST.	5-HT ↑, NE ↑, and 5-HIAA/5-HT ↓ in both hippocampus and frontal cortex.	Neurotransmitters modulation	(Chen et al., 2015)
Ferulic acid	40 and 80 mg/kg	Reserpine	Male ICR mice	The immobility time ↓ in TST and FST.<br>Thermal pain threshold ↑.	5-HT ↑, NE ↑, and DA ↑ in brain.	Neurotransmitters modulation	(Xu et al., 2013)
Ferulic acid	40 and 80 mg/kg	Reserpine	Male ICR mice	The immobility time ↓ in FST.<br>Mechanical pain threshold ↑.<br>Thermal pain threshold ↑.	5-HT ↑, NE ↑, and SOD ↑ in both hippocampus and frontal cortex.	Neurotransmitters modulation<br>Neuroprotection	(Zhang et al., 2013)

Drug	Dosage	Inducement	Animal	Behavioral test after treatment	Molecular mechanism after treatment	Pharmacological effects	Reference
Ferulic acid	0.01 mg/kg	TST	Male Swiss mice	The immobility time ↓ in TST.	Activated PKA, CaMKII, PKC, MAPK/ERK or PI3K/Akt signaling pathways		(Zeni et al., 2012)
Ferulic acid	25 and mg/kg	FST	Male SD rats	The immobility time ↓ in TST.<br>The crossings ↑ in OFT	5-HT ↑, ACTH ↓, CRH ↓, and Ghrelin ↑ in both plasma, hippocampus, and prefrontal cortex.	Neurotransmitters modulation<br>HPA axis regulation	(Zhang et al., 2011)
Ferulic acid	50, 100 and 250 mg/kg	CORT	Male ddY mice	The immobility time ↓ in FST.	BrdU-positive cells ↑, pCREB-positive cells ↑, and BDNF ↑ in DG.	Neurogenesis promotion	(Yabe et al., 2010)
Berberine	5 and 10 mg/kg	CUMS	Male C57BL/6J mice	The sucrose preference ↑ in SPT.<br>The immobility time ↓ in TST and FST.	Trim65 ↓, NLRP3 ↓, Cleaved caspase-1 ↓, ASC ↓, GSDMD-N ↓, pro-IL-1β↓, IL-1β↓, pro-IL-18 ↓, IL-18 ↓, TNF-α ↓, NLRP3/Iba-1 positive cells ↓, Iba-1 positive cells ↓, and Nissl-positive cells ↑ in hippocampus.	Anti-neuroinflammation<br>Neuroprotection	(Yang et al., 2023)
Berberine	100 and 200 mg/kg	CORT	male C57BL/6N mice	The sucrose preference ↑ in SPT.<br>The immobility time ↓ in TST and FST.<br>The distance ↑ and crossing ↑ in OFT.	TNF-α ↓, IL-1β ↓, IL-6 ↓, and IL-10↓ in both serum, prefrontal cortex, and hippocampus.<br>PSD95 ↑, SYN ↑, BrdU and DCX positive cells ↑, Spine density ↑, mushroom spines ↑, thin spines ↑, depth of post synaptic dendrites ↑, and length of post synaptic dendrites ↑ in hippocampus.<br>Frequency of mEPSC ↑ and frequency of sEPSC ↑ in medial prefrontal cortex	Neurogenesis promotion<br>Anti-neuroinflammation<br>Synaptic plasticity improvement	(Qin et al., 2023)
Berberine	50 and 100 mg/kg	CUMS	Male Wistar rats	The sucrose preference ↑ in SPT.<br>The immobility time ↓ in FST.<br>The distance ↑ and rearing ↑ in OFT.	5-HT ↑, NE ↑, DA ↑, and BDNF ↑ in hippocampus.<br>Firmicutes ↑, Lachnospiraceae ↑, and	Neurotransmitters modulation<br>Gut microbiota alternation	(Huang et al., 2023)

Drug	Dosage	Inducement	Animal	Behavioral test after treatment	Molecular mechanism after treatment	Pharmacological effects	Reference
					Bacteroidetes ↓ in Gut microbiota essay.<br>Total SCFAs ↓, acetic acid ↓, propanoic acid ↓, and Isovaleric acid ↑ in fecal samples.		
Berberine	2.5, 5, and 10 mg/kg	CUMS	Male C57BL/6 mice	Total distance ↑, central distance ↑, total walking time ↑, and central walking time ↑ in OFT.<br>The immobility time ↓ in FST.<br>The latency ↓ in NSFT.	NeuN ↑, SYN1 ↑, and PSD-95↑ in DG and CA3.<br>TNF-α ↓, IL-4 ↓, IL-10 ↑, and IL-1β ↑ in serum.<br>TNF-α ↓, IL-4 ↓, IL-1β ↓, IL-10 ↑, 5-HT ↑, DA ↑, GABA ↑, Glu ↓ and Ach ↓ in hippocampus.<br>5-HT ↑, DA ↑, GABA ↑, Glu ↓ and Ach ↓ in cortex.<br>5-HT ↑, GABA ↑, Glu ↓ and Ach ↓ in striatum.<br>5-HT ↑, DA ↑, and GABA↑ in amygdala.<br>Tryptophan biotransformation into serotonin ↑, TPH1 ↑, kynurenine metabolism pathway ↓ and IDO1 ↓ in hippocampus metabolomics.	Anti-neuroinflammation<br>Synaptic plasticity improvement<br>Neurotransmitters modulation<br>Neuroprotection	(Ge et al., 2023)
Berberine	25, 50, and 100 mg/kg	CUMS	Male ICR mice	The sucrose preference ↑ in SPT.<br>The crossing ↑, grooming ↑, and distance↑ in OFT.<br>The immobility time ↓ in FST and TST.<br>The latency ↓ in NSFT.	KYN ↓, KYN/TRP ↓, 5-HT ↑, 5-HTAA ↑, 5-HT/5-HTAA ↑, IDO1 ↓, MAOA ↓, and DDC ↑ in hippocampus.	Neurotransmitters modulation<br>Neuroprotection	(Wang et al., 2022b)
Berberine	20 mg/kg	CUMS	Male C57BL/6J mice	The sucrose preference ↑ in SPT.<br>The immobility time ↓ in FST.	BDNF ↑, miR-34b-5p ↓, and miR-470-5p ↓ in hippocampus.	Neuroprotection	(Zhan et al., 2021)
Berberine	100 mg/kg	CUMS	Male C57BL/6J mice	The sucrose preference ↑ in SPT.<br>The latency ↓ in NSFT.	miR-34a ↓, SYN1 ↑, Bcl-2 ↑, DCX positive cells ↑, and dendritic spine ↑ in hippocampus.	Neuroprotection<br>Neurotransmitters modulation<br>Neurogenesis promotion	(Yi et al., 2021)
Berber	50, 100,	CUMS	Male	The sucrose preference ↑ in SPT.	AMPK ↑, ATP ↑, lactic acid ↓, and pyruvate ↓ in	Neuroprotection	(Lu et al., 2021)

Drug	Dosage	Inducement	Animal	Behavioral test after treatment	Molecular mechanism after treatment	Pharmacological effects	Reference
ine	200 mg/kg		ICR mice	The immobility time ↓ in FST.	hippocampus.		
Berberine	20 mg/kg	CORT	Male C57BL/6 mice	The sucrose intake ↑ in SPT	NMDAR ↑, AMPAR ↑, BDNF ↑, and CREB ↑ in hippocampus.	Neuroprotection	(Gong et al., 2019)
Berberine	50 mg/kg	CFA	Male ICR mice	The immobility time ↓ in FST.<br>The latency ↓ in NSFT.<br>Thermal pain threshold ↑.<br>Mechanical pain threshold ↑.	CCL2 ↓, IL-1β↓, IL-6 ↓, and TNF-α ↓ in spinal.	Anti-neuroinflammation	(Xu et al., 2018)
Berberine	50, and 100 mg/kg	CUMS	Male ICR mice	The sucrose preference ↑ in SPT.<br>The latency ↓ in NSFT.	TNF-α↓, IL-6 ↓, IL-1β ↓, CD11b ↓, p-NF-κB/NF-κB ↓, p-IKKα/IKKα ↓, p-IKKβ/IKKβ ↓, and iNOS ↓ in hippocampus.	Anti-neuroinflammation	(Liu et al., 2017b)
Berberine	5, and 10 mg/kg	OVX	Female ICR mice	The immobility time ↓ in FST.	pCREB/CREB ↑ in frontal cortex.<br>BDNF ↑, peEF2/eEF2 ↓, and c-Fos ↑ in hippocampus.	Neuroprotection	(Fan et al., 2017)
Berberine	40, and 200 mg/kg	CUMS	Male SD rats	The sucrose preference ↑ in SPT.<br>The immobility time ↓ in FST.<br>The traversing ↑, vertical time ↑, and grooming ↑ in OFT	reverse the physical damage brought about by stress within the gastric mucosa and intestinal microvilli of the stomach, ileum, cecum and colon.		(Zhu et al., 2017)
Berberine	50 and 100 mg/kg	CORT	Male ICR mice	The sucrose preference ↑ in SPT.<br>The immobility time ↓ in FST.	CORT ↓ in serum.<br>BDNF ↑ in hippocampus.	HPA axis regulation<br>BDNF increasement	(Shen et al., 2016)
Berberine	10, 20, and 50 mg/kg	Morphine	Male SD rats	The immobility time ↓ in FST.<br>The open arms time ↑ and open arms frequency ↑ in EPM.	CRF ↓ in hypothalamus.<br>BDNF ↑ in hippocampus.	HPA axis regulation<br>BDNF increasement	(Lee et al., 2012)
Timosa	50, 100,	FST & TST	Male	The immobility time ↓ in FST and TST.	5-HT, NE, and DA reuptakes↓ in brain.	Neurotransmitters modulation	(Lu et al., 2010)

Drug	Dosage	Inducement	Animal	Behavioral test after treatment	Molecular mechanism after treatment	Pharmacological effects	Reference
ponin B-II	and 150 mg/kg		ICR mice				
Mangiferin	10, 50, and 100 mg/kg	CUMS+tumor bearing	Male BALB/c mice	The immobility time ↓ in FST and TST.<br>The social interaction ↑ in SIT	TNF-α↓, IL-6 ↓, and IL-1β ↓ in serum.	Anti-neuroinflammation	(Tao et al., 2023)
Mangiferin	20 and 60 mg/kg	PDD	Female BALA/c mice	The immobility time ↓ in FST and TST.<br>The latency ↓ in NSFT.	Iba-1 ↓, TNF-α↓, IL-6 ↓, and IL-1β ↓ in hippocampus.	Anti-neuroinflammation	(Yan et al., 2022)
Mangiferin	20, 40, and 80 mg/kg	CUMS	Male SD rats	The sucrose preference ↑ in SPT.<br>The immobility time ↓ in FST.<br>The crossing ↑ in OFT.	NLRP3 ↓, ASC ↓, Caspase-1 ↓, IL-18 ↓, IL-1β↓, IL-6 ↓, and TNF-α ↓ in hippocampus.	Anti-neuroinflammation	(Zhang et al., 2021b)
Mangiferin	20 and 40 mg/kg	CMS	Male ICR mice	The sucrose preference ↑ in SPT.<br>The immobility time ↓ in FST and TST.<br>The distance. ↑, rearing ↑, central frequency ↑, and central time ↑ in OFT.	CORT ↓ in serum.<br>NLRP3 ↓, ASC ↓, Caspase-1 ↓, IL-18 ↓, IL-1β↓, IL-6 ↓, and TNF-α ↓ in hippocampus.	Anti-neuroinflammation	(Cao et al., 2017)
Mangiferin	40 mg/kg	CORT	Male Swiss albino mice	The immobility time ↓ in FST and TST.	MDA ↓, nitrite ↓, GSH ↓, BDNF ↑, IL-1β↓, TNF-α ↓, IDO ↓, and KYN/TPH1 in hippocampus.	Anti-neuroinflammation<br>Neuroprotection	(Luo et al., 2017)
Mangiferin	20 and 40 mg/kg	LPS	Male Swiss mice	The sucrose preference ↑ in SPT.<br>The crossing ↑, rearing ↑, and immobility ↓ in OFT.<br>The immobility time ↓ in FST and TST.<br>The social interaction time ↓ in SIT.	SOD ↑, CAT ↑, reduced glutathione ↑, MDA ↓, nitrite ↓, and IL-1β ↓ in hippocampus and prefrontal cortex.<br>BDNF ↑ in hippocampus.	Anti-neuroinflammation<br>Neuroprotection	(Jangra et al., 2014)

Drug	Dosage	Inducement	Animal	Behavioral test after treatment	Molecular mechanism after treatment	Pharmacological effects	Reference
Mangiferin	25, 50, and 100 mg/kg	CUMS	Male Kunming mice	The crossing ↑and distance ↑ in OFT.<br>The immobility time ↓ in TST.	BDNF ↑ in hippocampus.	BDNF increasement	(Fu et al., 2013)

Abbreviations: 5-HIAA, 5-hydroxyindole acetic acid; 5-HT, 5-hydroxytryptamine; 5-HTR1A, 5-hydroxytryptamine receptor 1A; Ach, acetylcholine; ACTH, adrenocorticotropic-hormone; Akt, protein kinase B; AMPAR, α-amino-3-hydroxy-5-methyl-4-isoxazolepropionic acid receptor; ASC, apoptosis-associated speckle-like protein; ATP, adenosine triphosphate; Bcl-2, B-cell lymphoma-2; BDNF, brain-derived neurotrophic factor; BIP, immunoglobulin heavy chain binding protein in pre-B cells; BrdU, 5-Bromodeoxyuridinc; CaMKII, calcium-dependent protein kinase II; CAT, catalase; CCL2, C-C motif chemokine ligand 2; CFA, complete freund's adjuvant; CMS, chronic mild stress; CORT, corticosterone; CREB, cAMP-response element binding protein; CRF, corticotropin releasing hormone; CRFBP, CRF binding protein; CRMP2, collapsin response mediator protein 2; CSDS, chronic social defeat srtress; CSF, cerebrospinal fluid; CUMS, chronic unpredictable mild stress; DA, dopamine; DCX, doublecortin; DDC, dopamine decarboxylase; DG, dentate gyrus; EAAT2, excitatory amino acid transporter; EGFR, epidermal growth factor receptor; EPM, elevated plus maze; ERK, extracellular signal-regulated protein kinase; FKBP5, FK506 binding protein 5; FST, forced swimming tests; GABA, gamma-aminobutyric acid; GFAP, glial fibrillary acidic protein; Glu, glutamate; GR, glucocorticoid receptor; GSDMD-N, gasdermin D; GSH-Px, glutathione peroxidase; HMGB1, high-mobility group box 1; HPA, hypothalamic-pituitary-adrenal; Iba-1, ionized calcium bindingadaptor molecule-1; IDO, indoleamine-2,3-dioxygenase; IL-10, interleukin-10; IL-18, interleukin-18; IL-1β, interleukin-1β; IL-4, interleukin-4; IL-6, interleukin-6; iNOS, inducible nitric oxide synthase; LPS, lipopolysaccharides; IκB, inhibitor of NF-κB; KYN, kynurenine; LH, learned helplessness; MAOA, monoamine oxidase A; MAP-2, microtubule associated protein-2; MAPK, mitogen-activated protein kinase; MDA, malondialdehyde; mGluR1, metabotropic glutamate receptor 1; mGluR5, metabotropic glutamate receptor 1; MS-RS, maternal separation combining chronic restraint stress; mTOR, mechanistic target of rapamycin; NE, norepinephrine; NeuN, neuronal nuclei; NF-κB, nuclear factor kappa-B; NLRP3, NOD-like receptor thermal protein domain associated protein 3; NMDAR, N-methyl-D-aspartate receptor; nNOS, neuronal nitric oxide synthase; NO, nitric oxide; Nr3c1, nuclear receptor subfamily 3 group C member 1; NSFT, novelty suppressed feeding test; OFT, open field test; OVX, ovariectomized; PI3K, phosphatidylinositide 3-kinases; PKA, protein kinase A; PKC, protein kinase C; PPD, perimenopausal depression; PRS, prenatal restraint stress; PSD95, post-synaptic density protein 95; RAGE, receptor for advanced glycation end-products; SGK, glucocorticoid-inducible kinase; SIRT6, SIT, social interaction test; sirtuin 6; SOD, superoxide dismutase; SPT, sucrose preference test; SYN, synaptophysin; SYN1, synaptotagmin1; TNF-α, tumor necrosis factor-α; TPH1, tryptophan 5-hydroxylase 1; Trim65, tripartite motif-containing protein 65; TrkB, tropomyosin related kinase B; TRP, tryptophan; TST, tail suspension test; XBP1s, X-box binding protein 1 spliced.
